# Microenvironmental genomic alterations reveal signaling networks for head and neck squamous cell carcinoma

**DOI:** 10.1186/2043-9113-1-21

**Published:** 2011-08-02

**Authors:** Gurkan Bebek, Mohammed Orloff, Charis Eng

**Affiliations:** 1 Genomic Medicine Institute, Cleveland Clinic, 9500 Euclid Avenue, Mailstop NE-50 Cleveland, OH 44195, USA; 2 Taussig Cancer Institute, Cleveland Clinic, 9500 Euclid Avenue, Mailstop NE-50 Cleveland, OH 44195, USA; 3 Case Center for Proteomics and Bioinformatics, Case Western Reserve University, 10900 Euclid Ave. Cleveland OH 44106, USA; 4 Case Comprehensive Cancer Center, Case Western Reserve University, 10900 Euclid Ave. Cleveland OH 44106, USA; 5 Department of Genetics, Case Western Reserve University, 10900 Euclid Ave. Cleveland OH 44106, USA

## Abstract

**Background:**

Advanced stage head and neck squamous cell carcinoma (HNSCC) is an aggressive cancer with low survival rates. Loss-of-heterozygosity/allelic imbalance (LOH/AI) analysis has been widely used to identify genomic alterations in solid tumors and the tumor microenvironment (stroma). We hypothesize that these identified alterations can point to signaling networks functioning in HNSCC epithelial-tumor and surrounding stroma (tumor microenvironment).

**Results:**

Under the assumption that genes in proximity to identified LOH/AI regions are correlated with the tumorigenic phenotype, we mined publicly available biological information to identify pathway segments (signaling proteins connected to each other in a network) and identify the role of tumor microenvironment in HNSCC. Across both neoplastic epithelial cells and the surrounding stromal cells, genetic alterations in HNSCC were successfully identified, and 75 markers were observed to have significantly different LOH/AI frequencies in these compartments (p < 0.026). We applied a network identification approach to the genes in proximity to these 75 markers in cancer epithelium and stroma in order to identify biological networks that can describe functional associations amongst these marker-associated genes.

**Conclusions:**

We verified the involvement of T-cell receptor signaling pathways in HNSCC as well as associated oncogenes such as *LCK *and *PLCB1*, and tumor suppressors such as *STAT5A, PTPN6, PARK2*. We identified expression levels of genes within significant LOH/AI regions specific to stroma networks that correlate with better outcome in radiation therapy. By integrating various levels of high-throughput data, we were able to precisely focus on specific proteins and genes that are germane to HNSCC.

## Background

HNSCC is the sixth most common cancer and remains a major cause of cancer morbidity and mortality worldwide [1]. More than 85% of head and neck squamous cell carcinomas (HNSCC) are related to tobacco use, while others may have a relationship to viral etiologies such as human papillomavirus (HPV) infection/colonization. Nevertheless, advanced stage HNSCC remains an aggressive cancer with low survival rates. Molecular studies suggest that HNSCC results from cumulative epigenetic and genetic alterations [2-4]. Various genomic regions and/or genes have been correlated with survival in HNSCC or classified as early detection/aggressiveness markers [2]. Albeit incomplete, such baseline knowledge of HNSCC genetics builds a foundation for exploration of functional associations between these structural alterations and tumorigenesis. Identifying such networks through a more systematic examination of HNSCC is a challenge and the focus of this study.

Recent genome-scanning technologies uncovered an unexpectedly large amount of structural variation (SV) in the human genome [2,5-9]. Structural variations comprise a large set of alterations including deletions, duplications, large-scale copy-number variants, inversions and translocations in the genome [10]. On the extreme, cancer genomes are known to attain frequent alterations in their gross chromosomal structure by amplification, deletion, translocation and/or inversion of chromosomal segments [11]. These structural variations can inactivate genes, produce multiple copies of genes thereby increasing gene activity or, in rare situations, result in the fusion of two genes. Alterations in tandem may be critical to cancer onset and progression.

Loss-of-heterozygosity/allelic imbalance (LOH/AI) scanning has been widely used to identify genetic alterations in tumor samples. The absence or an imbalanced signal of a DNA marker in the tumor sample would suggest LOH/AI in these cancerous cells [12]. Numerous studies reporting localized and/or genome-wide LOH/AI analyses have discovered specific loci with consistently high frequencies of LOH/AI in HNSCC. These observations have provided key clues for identification of tumor suppressor genes in this malignancy [2,13]. Moreover, it is now common practice to utilize laser capture micro-dissection (LCM) and LOH/AI analysis of tumor compartments, namely, neoplastic epithelial cells and the surrounding cancer-associated (previously presumed to be non-cancerous) stromal cells (part of the tumor microenvironment) [14-21]. For example, LOH/AI analysis of DNA from the neoplastic epithelial cells of invasive breast carcinomas and surrounding stroma revealed that stromal somatic mutations of *TP53 *in stromal cells, but not epithelial neoplasia, correlated with regional nodal metastases [22]. In the absence of stromal *TP53 *mutation, LOH/AI at 5 specific loci in the stromal cells also correlated with regional nodal metastases [22]. Subsequently, only with extensive empiric molecular and cell biology studies did a mechanism for this genetic observation emerge [23]. In general, however, extended functional associations of genes within these regions with their cellular signaling mechanisms have yet to be made. It is hoped that the approach described here will minimize the time and effort put forward for pinpointing functional mechanisms from tumor-associated bicompartmental somatic genomic observations without prolonged repeated empiric work on multiple candidate pathways.

In this study, therefore, we have applied an integrated network discovery framework [24-26] to identify distinct signaling pathway networks (SPN) of the two compartments of HNSCC. Genes of interest are surveyed and signaling networks identifying genes affected by these variations are visualized. We also investigated bicompartmental genomic alterations and their associated SPN's in the context of radiation therapy and human papilloma virus (HPV) status, both germane factors in HNSCC treatment response. Ultimately, our systems biology approach of pathway identification should provide invaluable knowledge in understanding the inter-compartmental and inter-network-based events in HNSCC tumorigenesis and importantly, guide empiric molecular and cellular biology experiments in a targeted manner.

## Results

**Figure 1:**
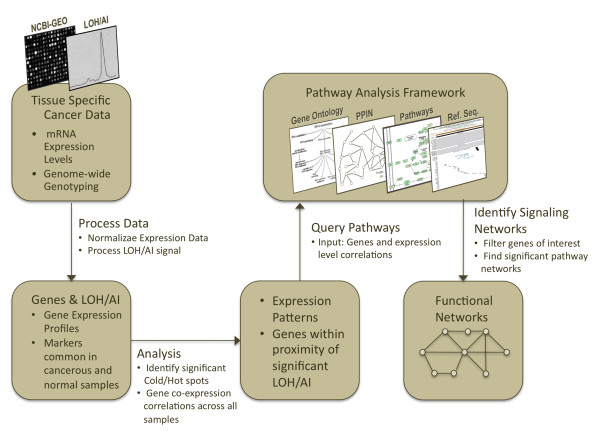
**Workflow for high-throughput data integration to help understand the molecular basis of cancer**. An integrative -omics signaling network identification process workflow that begins with processing tissue specific data (instrument outputs). Microarray data is normalized to make comparisons of expression levels and transformed to select genes for further analysis. LOH/AI signals are analyzed to identify regions (and hence regional genes) for both tumor and normal tissue (or noncancerous cells). Next, genes observed within proximity of these markers are merged with their corresponding microarray probes to create expression profiles. In this analysis step, expression profiles are used to calculate Pearson's coexpression correlations among gene pairs. These results are fed into the Pathway Analysis Framework. Integrating gene-gene coexpression values, annotations from Gene Ontology, known signaling pathwas, protein sequence information, protein-protein interaction networks, and protein subcellular colocalization data, pathways are predicted and filtered. Significant pathway subnetworks are merged to form signaling networks connecting genes of interest. The networks and structural variations identified are put together to create a descriptive functional network, creating a molecular basis for the cancer studied. This type of workflow, which we utilized, can be applied to using integrative systems biology approaches to study cancer and other pathologies.

To identify signaling pathway networks for HNSCC stroma and epithelium compartments, we devised a computational workflow in which we integrated our own empirically-derived LOH/AI analysis of genomic DNA from epithelial and stromal compartments of 122 HNSCC specimens [16] with publicly available HNSCC-derived genome-wide genomic and functional-genomic datasets and high-throughput proteomics and cellular data (Figure 1). In this approach, we processed large-scale genome-wide scans of HNSCC tumors to generate a list of candidate genes. This list is then used to search for likely HNSCC-relevant signaling pathways in the pathway analysis framework (based on [24-26]).

### LOH/AI gene identification

Genotyping of 366 microsatellite markers of both epithelium and stroma samples from the 122 patients' HNSCC tumors (Table 1) revealed 75 marker locations as significant for frequent genomic alterations. This set of 75 markers was examined in this study. LOH/AI regions that have significantly higher frequencies of LOH/AI compared with other markers along the same chromosome are defined as *hot spots*, as previously operationally defined via a model-based approach [16,22]. Regions that have significantly lower frequencies of LOH/AI compared with other markers along the same chromosome are termed *cold spots *(See Additional File 1, Table S1 and Table S2 for a complete list of markers). The hot and cold spots identified [in either compartment] are approximately equal in number (37 hot spots vs. 34 cold spots [See Table 2]). However, the number of hot/cold spots (hot spots + cold spots) identified only in stroma is about three-fold compared to those identified in the epithelium. In addition to these 71 markers to be brought forward for integration with other platforms, we also included four more markers that we previously found to correlate with tumor size (one from stroma) and regional nodal status (two from stroma and one from epithelium) in HNSCC in this set [16].

**Table 1:** Patient Characteristics (N = 122)*

Characteristic	Frequency, No. (%)
Sex	
Men	86 (71.1)
Women	35 (28.9)
Age, mean (SD), y	58.5 (12.9)
Primary site	
Oral	55 (46.6)
Pharynx	63 (53.4)
Stage	
I	16 (14.5)
II	22 (200)
III	34 (30.9)
IV	38 (34.5)
Tumor Size	
T1	23 (20.9)
T2	44 (40.0)
T3 or T4	43 (39.1)
Regional nodal metastases	
N0	44 (39.3)
N1	24 (21.4)
N2	39 (34.8)
N3	5 (4.5)
Grade	
Low G1 or 2	83 (80.6
High G3	20 (19.4)

* Data were not available for all patients.

Using laser capture microdissection, epithelium and stromal tissue compartments of squamous cell cancer lesions of head and neck from 122 samples were acquired.

**Table 2:** Hot spot-, cold spot- and clinicopathological feature-associated microsatellite markers identified in HNSCC tissue compartments

Compartment	LOH/AI	Markers
Epithelium and stroma	Hot spots	5
	
	Cold spots	6

Epithelium only	Hot spots	10
	
	Cold Spots	7
	
	CPF	1

Stroma only	Hot spots	22
	
	Cold spots	21
	
	CPF	3

	Total	75

Using laser capture microdissection, epithelium and stromal tissue compartments of squamous cell cancer lesions of head neck from 122 samples were acquired. 366 microsatellite markers were used to identify significantly higher/lower frequency of LOH/AI at a marker or markers compared with other markers along the same chromosome. The table shows the number of cold spot, hot spot or clinicopathological features (CPF) identified in epithelium only, stroma only, and both in epithelium and stroma.

For this set of 75 markers, we extended marker locations 250 kb in both directions of a marker to identify genes within proximity. The parameter (250 kb) was chosen for computational flexibility. This extension returned 273 genes that lay within proximity of these marker locations (See Additional File 1, Table S3). The number of genes included in the region increases linearly as the flanking regions are extended (See Additional File 2, Supp. Text). A larger set of genes diminishes the effectiveness of the methodology since the number of unrelated genes increases. For instance, if genes within the same loci of identified markers are used (> 250 kb, varying based on loci size), the mapping would return ~2200 genes for these 75 markers. Thus, we decided against this all-encompassing approach so that we could establish an effective methodology (See Additional File 2, supplementary text and Additional File 3, Figure S4).

### Genes are filtered by establishing networks

**Figure 2:**
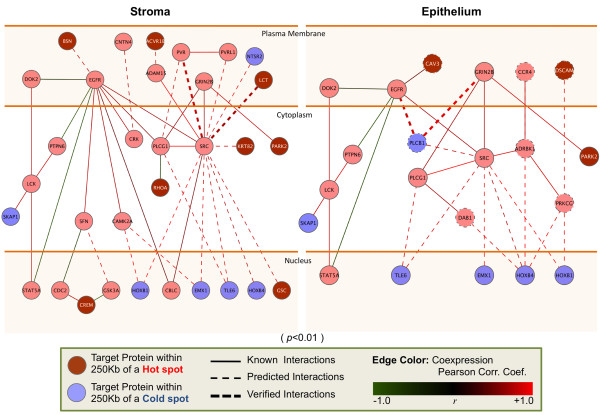
**The signaling pathway networks**. Networks are generated for stroma only and epithelium only with p < 0.01. The proteins (represented by nodes) are placed in their intra-cellular localization, with the plasma membrane represented at top and the nucleus at the bottom. The nodes are colored blue if they are within 250 kb of an identified cold spot and red if they are within 250 kb of an identified hot spot. Pink nodes represent the intermediary proteins identified through the computational framework. The interaction colors represent the Pearson's correlation coefficient (*r*) of the two neighboring proteins' mRNA levels. If the edge is colored red, the two proteins have a positive mRNA expression correlation, whereas green represents the opposite. The solid edges show known interactions, while the dashed edges are interactions predicted via homology/family information [24]. The predicted interactions are bolded if they are verified with independent studies through a literature search.

The pathway identification framework utilizes various datasets including mRNA gene expression profiles, tissue-specific genotyping data, protein-protein interactions, protein subcellular localization data, and functional annotations of genes (Gene Ontology [27]), to connect genes within proximity of the 75 significant LOH/AI marker locations in a signaling network (see Methods for a brief description). For calculating associations, genes not linked to HNSCC through this integration step were dropped from further consideration. The remaining gene list from the 75 marker regions was divided into two subsets according to their subcellular compartment (See Additional File 1, Table S4 and Table S5 for a complete list of epithelium and stroma markers and genes used). First, a global protein-protein interaction network was built by integrating these data sources. Next, gene lists from these marker locations were utilized to search for networks that are specific to the two compartments. In this framework, the interaction network was queried for signaling proteins connected to each other on a linear path (pathway segments). Using these signaling chains acquired in the search process (p-value < 0.01, please see supplementary methods for details), signaling pathway networks from the stroma and epithelium were generated (Figure 2). These networks depict significant signaling events that occur in the two compartments. Signaling events such as T-cell signaling, EGFR-PTK2B signaling, and interactions between various tumor suppressors and oncoproteins were identified which shape the set of filtered genes (see Discussion for extended analysis).

### Signaling networks highlight functional associations of tumor related genes

Signaling events in the cell play a critical role in the execution of key biological functions. To further investigate the role of the filtered genes within LOH/AI regions of interest, we searched for signaling pathway networks, which were generated using mRNA expression levels, known key signaling pathways, protein-protein interactions, and characteristics of these proteins. The signaling pathway search greatly decreased the number of genes associated with each marker (down ~50 from 273). In this way, an extended list of genes was reduced to a short list of genes that are functionally correlated with one another in the HNSCC context.

We then compared our short list of genes with the oncogenes and tumor suppressor genes that have been previously identified to be associated with HNSCC and other cancers in earlier studies (listed in Table 2). Earlier studies may have identified genes of interest by observing their structural loss or reduction in function. In the present work, our network search workflow (Figure 1) has been successfully verified by identifying genes that were previously associated with HNSCC. For further verification, we found that our methodology accurately identified genes that were previously identified as tumor suppressors, proto-oncogenes and metastasis-related genes in earlier studies (see Additional File 1, Table S6, Table S7 and Table S8). In addition, the generated networks included known head and neck cancer biomarkers. Common fragile site genes (DAG1 and PARK2) and various genes that have elevated expression patterns in cancers are also connected in these networks.

### Structural variations are involved in initiation and progression of HNSCC

**Figure 3:**
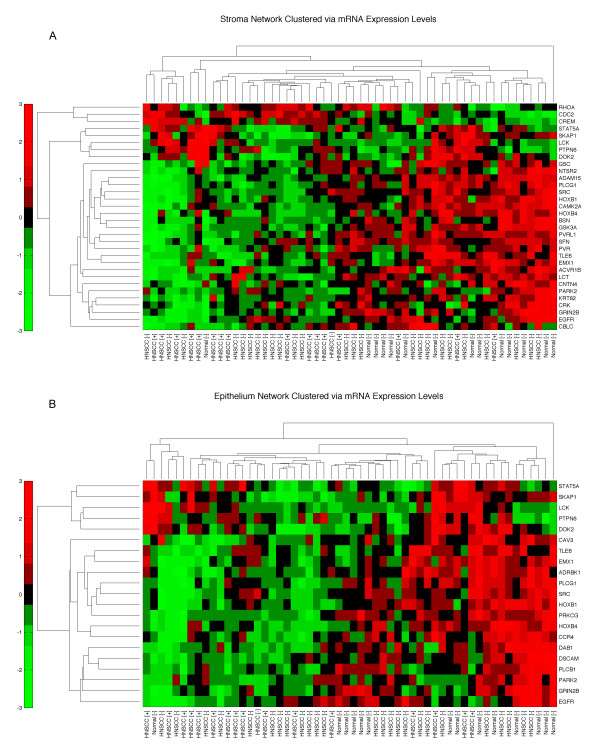
**Stroma network clustered via mRNA expression levels**. mRNA expression levels of (A) Epithelium and (B) Stroma network proteins are clustered using hierarchical clustering. Horizontal clustering depicts genes and vertical clustering groups tumors through expression levels. The labels below represent HNSCC tumors with HPV status (+ or -) and normal tissue included in the mRNA expression microarray study [47].

Listed in Table S9 are genes that likely have structural variations, i.e., hot spot marker genes that harbor gene gain or loss in HNSCC. Transcriptional profiles of HNSCC using both stroma and epithelium network-associated genes were hierarchically clustered to show that we have acquired networks depicting the connections of HNSCC aberration sites. In other words, we wished to see if the likely alteration of genes in these networks is also altering functionality of these genes in cancers. We also observed that most network-associated genes are consistently turned off, and only a small number of genes have increased expression, such as *RHOA, CDC2*, and *CREM *in stroma (Figure 3).

In summary, networks signifying medium- to large-scale structural variations are predicted through integration of genome-wide LOH/AI analysis, tumor-derived mRNA expression levels, and high throughput proteomic, pathway and annotation data.

### Verifying networks identified via cluster analysis of mRNA expression data

To strengthen our claim that the generated networks are highly significant in describing the disease, in this case HNSCC, we also analyzed randomly picked genes from the protein-protein interaction database, the primary database of the computational framework. We acquired microarray expression levels from the same mRNA dataset for these genes and generated an unsupervised clustering for these genes for comparison. These clusters show increased disarray (See Additional File 3, Figure S2) when compared to the expression patterns of the genes placed in the network through the computational framework.

**Figure 4:**
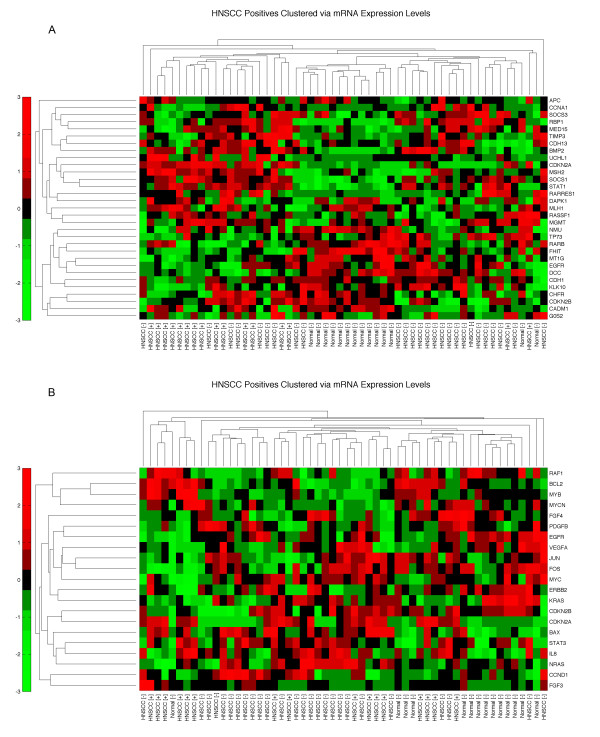
**HNSCC-associated genes cluster via mRNA expression levels**. A: PubMeth (Reviewed methylation database of cancer genes, [28]) genes associated with HNSCC B: Literature surveyed HNSCC associated genes are clustered using the mRNA expression profiles to show classification power of earlier HNSCC studies compared to genes identified through the network framework.

**Figure 5:**
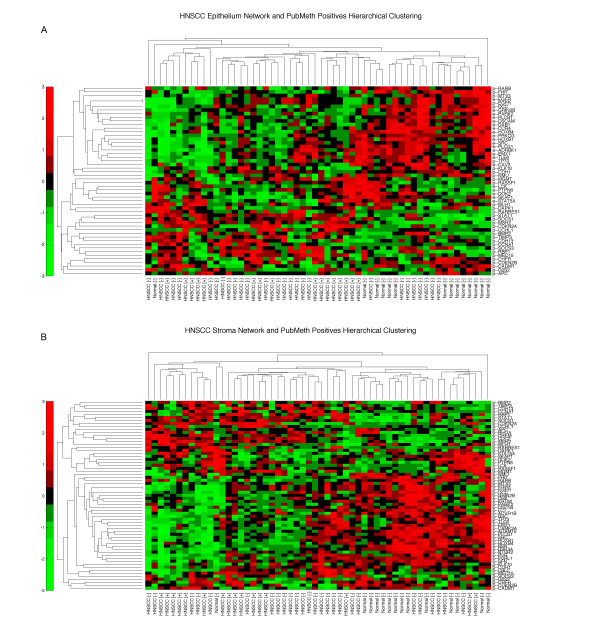
**(A) Epithelium and (B) Stroma network and methylated genes clustered via mRNA expression levels**. PubMeth (Reviewed methylation database of cancer genes, [28]) genes associated with HNSCC are marked with 'x', and network genes are marked with '+'. The heatmaps show corroboration of our findings with earlier studies, hence strengthening our discovered networks [2,13,52].

Since we have observed that in a highly significant network, most genes have altered expression, we have also generated similar clustering with biased selections. We compiled lists of genes associated with HNSCC from PubMeth (Reviewed methylation database of cancer genes [28], Additional File 1, Table S9) and from literature search (Additional File 1, Table S10). Since these lists consist of genes that are positively correlated with HNSCC, we first generated unsupervised clustering of these listed genes utilizing the HNSCC mRNA expressional data in a similar fashion. We also merged each of these two lists with our network genes and re-clustered these genes. We observe that PubMeth genes can classify HPV+ and HPV- HNSCC and normal tissue mRNA expression profiles, while the literature scan gene list cannot deliver similar classification (Figure 4). However, the latter were still able to classify normal tissue versus HNSCC as a whole. The combined lists show that our findings are consistent with prior observations, thus supporting our network-based conclusions (Figure 5).

### Radiation response of identified genes and networks

We have further analyzed the above identified HNSCC epithelium- and stroma-associated network genes (Figure 2) using Global Test [29]. Global Test is a statistical test that can be used to identify association between the expression profile of groups of genes and a given outcome. In this case, we utilized this statistic to extract causal gene clusters that correlate with resistance or sensitivity to radiation exposure, which may be viewed as an important form of genotoxic stress. For these purposes, we have included seven sets of genes, significant microsatellite marker-associated genes (Additional File 1, Table S4 and Table S5), the genes identified in stroma and epithelium networks (Additional File 1, Table S6 and Table S7), the intersection of the two networks, and only the hot/cold spot genes that were identified in these two networks. When genotoxic stress outcome (sensitive/resistant) was measured in head and neck tumor cell lines [30] (GSE9714) and NCI Anti-Cancer Drug Screen (NCI60) cell lines [31] (GSE7505), only the stroma-associated genes (Table 3 and Additional File 1, Table S5) were statistically significant using the global test statistics.

**Table 3:** Proteins in the signaling networks associated with structural hot- and cold-spots generated for stroma and epithelium of HNSCC

Category	Stroma	Epithelium
Tumor Suppressor Gene	*ACVR1B, DOK2, PARK2, PTPN6, STAT5A*	*DOK2, PTPN6, STAT5A*

Proto-oncogene	*CRK, **EGFR**, LCK, SRC*	***EGFR***, LCK, PARK2, SRC

HNC Biomarker	*SFN*	*CAV3*

Oncogenic when overexpressed	*RHOA*	

Increased expression in Cancers	*CDC2, CREM, HOXB4, NTSR2, PVR, PVRL1*	*ADRBK1, **CCR4**, HOXB4*

Inactive in Cancers		*DAB1*

Serine/threonine protein kinase	*CAMK2A, **GSK3A***	*PRKCG*

Role in Metastasis	*GSC, PLCG1*	*PLCB1, PLCG1*

HNSCC association not identified yet	*ADAM15, BSN, CBLC, CNTN4, EMX1, GRIN2B, HOXB1, KRT82, SKAP1, TLE6*	*DSCAM, EMX1, GRIN2B, HOXB1, SKAP1, TLE6*

The categories are determined by consensus literature terms. Genes whose products are studied as or utilized as drug targets are emboldened. Overall, 31 proteins in stroma and 21 for epithelium are identified through the signaling pathway search framework (Refer to Additional File 1 Table S6 and Table S7 for more details and references).

## Discussion

In this study, we sought to identify signaling pathway networks in HNSCC-derived carcinoma and their associated stroma by genotyping DNA from each compartment with microsatellite markers and integrating independent publicly available HNSCC-relevant somatic microarray-based mRNA expression and other datasets [32], resulting in a first description of integrative -omics-derived genes to signaling pathway networks in the neoplastic epithelial carcinoma cells and the surrounding tumor-associated stromal fibroblasts. We followed a computational workflow that integrates extensive amounts of high-throughput data, namely protein-protein interactions, gene ontology annotations, protein colocalization data, and known signaling pathways to form signaling networks that can lead to a better understanding of HNSCC (Figure 1). In addition, it is hoped that this type of approach would also result in specific pathways that can be targeted for empiric study linking genomic variation and pathogenesis without taking a candidate approach in functional analysis.

Genetic alterations such as copy number aberrations or LOH/AI have been shown to be associated with HNSCC initiation and progression [2]. In LOH/AI testing, the microsatellite markers are informative in a location-specific manner, however, these markers are an average of 9 cM apart. Hence, we extended the coverage to 250 kb flanking each side of the 75 significant (71 hot + cold spot markers + 4 markers associated with tumor size/nodal metastases) marker locations to generate a list of genes in close proximity. By choosing a shorter segment of the genomic region(s) near a significant marker (500 kb/marker instead of the marker's whole locus, in this case, 9 × 2 cM), we were able to narrow our search space and increase efficiency and minimizing false positives. We believe that our approach has identified significant genomic regions with viable functional associations. In this study, we have utilized microarray data that were generated from tumor samples that were at least 80% tumor cellularity [32]. An 80% tumor-cellularity does not mean 20% are stroma. Because we are looking at tumor-associated stroma, the 80% tumor-cellularity should also contain its tumor-associated stromal cells, but the precise make-up is unknown. The lack of publicly available subcompartment-specific gene expression profiles certainly poses its own challenges. However, since the pathway analysis is seeded from the genomic alterations of the subcompartments, the microarray data should still carry general patterns of expression profiles from head and neck tissue. Hence, the resulting pathways so identified should represent reasonably accurate stroma- and epithelium-specific signaling pathway networks. The generated networks contain a significant number of stroma- and epithelium-specific genes identified through the genotyping experiments. The networks reflect this classification via utilization of an integrative -omics approach. This reduces any false signals that might be introduced via any platform that is utilized.

### Signaling pathways of HNSCC

In this study, identifying large numbers of frequent LOH/AI in stroma suggests that genetic alterations in this compartment of the tumor precede the genetic alteration in the surrounding cells, which might be consistent with the well-known field-effect theory of cancerization [33,34]. We report networks based on these significant markers, which also highlight hot spot marker genes that mostly cluster in the stromal network. In recent studies, the interaction of epithelium and stroma of breast carcinoma was investigated [14,35,36]. Similar to our observation in HNSCC, the location of the LOH/AI regions in the epithelial cells of breast cancer are concentrated in a smaller region, specifically, a smaller number of markers with much higher LOH/AI frequencies; whereas in the stromal cells, they are more spatially complex, distributed over a larger number of loci. Using these observations, a model of carcinogenesis was developed, at least for breast cancers: transformation initiates in the epithelial cells (higher LOH/AI frequencies) while stromal genomic alterations may dictate biology and in term affect the epithelial component [14]. This genomic observation has been mechanistically validated [23].

We have observed a larger number of genetic alterations in the tumor stroma than in the epithelium. Additionally, not all changes in epithelium are observed in stroma. However, these added changes could play a different and parallel role in HNSCC carcinogenesis. This observation parallels the findings of independent studies showing somatic mutations and/or LOH/AI in the stroma of breast cancer [14,37] colon cancer [37], bladder cancer [38], and ovarian cancer [39]. It is plausible that the large number of markers involved in the stroma of HNSCC and bladder cancer could represent a field effect after exposure to shared carcinogens. Just as in other cancers such as those of the breast, the diversity of markers in the stroma may explain biological diversity [14]. The changes in the stroma resulting from alterations in the signaling pathways may then cross-talk with the cancer epithelium and induce genomic instability, as has been shown by Lisanti's group for breast cancers [23].

### Similarities and differences between stroma and epithelium networks

The identified signaling pathway networks point out differences in signaling in stroma and in epithelium (Figure 2). In investigating the expression correlations among proteins to identify the pattern of up/down regulation in tumors versus their corresponding normal tissues, *PARK2 *(Parkinson disease [autosomal recessive, juvenile] 2, parkin), a gene lying in a hotspot, was found to be common in both compartments' networks. *PARK2 *is within the fragile site *FRA6E *on chromosome 6, a region shown to be unstable and prone to breakage and rearrangements. *DAG1 *from this region was observed as inactivated in multiple cancers [40]. We observe that both of these genes in HNSCC are affected (Figure 2) with likely loss of expression.

*EGFR*-*PTK2B *signaling modulates ubiquitin (Ub)/proteasome pathway-mediated intracellular trafficking. *PYK2B *activation is also critical for the activation of *SRC *downstream of *EGFR*, which we do not observe in HNSCC. In this study, we observe that *EGFR *transactivation prevented the phosphorylation of the nonreceptor tyrosine kinases *PYK2B *and *SRC*, locating these kinases downstream of the transactivated *EGFR *as noted. Although as highlighted in the signaling networks, *SKAP1 *(*SRC *kinase associated phosphoprotein 1) is identified as a cold spot, the lack of signaling starting through *EGFR *prevents *SRC *activation. *SRC *is expressed at low levels in most cell types and, in the absence of appropriate extracellular stimuli, maintained in an inactive conformation.

We associated genes that are activated or have gain-of-function in other cancers with those found in HNSCC, e.g., *HOXB4, PVR, RHOA, ADRBK1 *and *CCR4*. Pathways that are common to multiple cancers can be identified by incorporating these types of oncogenes from multiple studies (Additional File 1, Table S6 and Table S7). Moreover, genes like *CCR4 *and *GSK3A *highlighted in this study are already candidate targets for therapy in other cancers [41]. Human breast, ovarian, renal, lung and colon tumor specimens have been analyzed for somatic *RHOA *mutations previously. No intragenic mutations in *RHOA *were found, nor a correlation between *RHOA *mRNA expression and the presence or absence of 3p21 deletions. This suggests likely duplication of *RHOA *in HNSCC as well (also verified by the increased mRNA expression levels shown in Figure 2) [42].

Regulatory T cells are important in modulating antitumor immune response. In both compartments, we see T-cell related signaling proteins, such as proto-oncogene *LCK (*T cell-specific protein-tyrosine kinase), tumor suppressor *PTPN6 *(Tyrosine-protein phosphatase), and *SKAP1 *(Src kinase-associated phosphoprotein 1). In cells, *SKAP1 *has a critical role in inside-out signaling (regulatory signaling that originate within the cell cytoplasm and are then transmitted to the external ligand-binding domain of a receptor) by coupling T-cell antigen receptor stimulation to the activation of integrins. In both compartments, *SKAP1 *interacts with *LCK*, which is most commonly found in T cells (Figure 2). In an earlier study [43], *STAT5B *was shown to contribute to *LCK*-induced cell proliferation and resistance to apoptosis. Similarly *STAT5A*, a *STAT5B *isoform, might be carrying out a similar activity in HNSCC. Hence, increased constitutive activation of *STAT5 *was detected in transformed compared with normal squamous cells. It is known that blockade of *TGF*-alpha or *EGFR*, ended *STAT5 *activation [44]. However, observing down regulation of *EGFR *in this cancer (Figure 2 and Figure 3), we conclude that the control on proliferation is lost.

### HNSCC biology is consistent in both HPV+ and HPV- patients

In this study, we did not observe differences in biological networks of HNSCC with and without human papillomavirus (HPV) in the context of the stroma. HPV infection is a strong risk factor for HNSCC [45] regardless of other factors such as tobacco or alcohol use. However, it should be noted that the HPV "effect" is germane only in certain oropharyngeal sites of HNSCC. Furthermore, depending on the manner and quantity of subtyping HPV, in fact, the jury is still out regarding the role of HPV in HNSCC. Unsupervised hierarchal clustering of mRNA expression levels of the network genes in stroma and epithelium showed that the expression patterns of HPV+ and HPV- patients are similar (Figure 3) in these networks. In essence, we hypothesize that these networks represent the biology in both tissue types, since they are built upon the genomic alterations and integrated with tissue specific message signals. Therefore, these heat maps, as opposed to subclustering into two patient groups (+ and -), reveal that only patient and normal sample differences are observed. Interestingly, when methylated genes are clustered via the same microarray data used in our network identification, the HPV+ and HPV - patients clearly separate from each other (Figure 4). To rule out that this might be due to tumor site differences, since HPV+ cases are observed with highest prevalence in oropharynx and base of tongue [46], we have repeated this clustering with a subset of patients that are site matched (11 pairs matched by site and stage), and observed a similar result (Figure S3).

The difference in methylated genes between HPV+ and HPV- patients is mostly due to changes in cellular machinery caused by either HPV's integration into the genome or the latter's inflammation-related effects on methylation. For instance *p16 *(*CDKN2A*) is known to be overexpressed in HPV+ HNSCC patients [13]. Although these differences were explained by HPV modifying the cellular expression machinery to create different expression profiles [47], our networks show that the essential genes in tumorigenesis are similar in both patient groups. In other words, HPV integration affects genes with similar downstream consequences as other mutational events observed in HPV- tumors. Hence, HNSCC-relevant pathways derived from LOH/AI regions of HNSCC as shown in our results (Figure 2, and Figure 3) has not been differentiated with HPV initiated HNSCC. Indeed, our current data suggest that whether it is HPV-associated or not, what is important are the final common pathways.

HNSCC is a result of cumulative genetic and epigenetic alterations [2]. In this study, we have only considered genetic markers of HNSCC and mRNA levels in the tissue to measure these changes. The computational data mining approach can be easily adapted to include any other high throughput experimentation, such as methylation profiles, single nucleotide polymorphisms etc. In future studies, using this successful workflow as a basis, we will extend our knowledgebase to fine map HNSCC signaling pathway networks.

### Stroma may mediate better response to radiation therapy

We have also investigated therapeutic predictive value of the networks identified. Utilizing human head and neck cancer tumor cell lines [30], the identified gene lists were subjected to a statistical test [29] to monitor their correlation with response to radiation therapy (see Methods). Expression of genes that are found within 250 kb on either side of each hot and cold spot LOH/AI markers in the stroma were statistically significant (Table 4; p-value = 0.029) when correlated with response to radiation therapy (151 genes were tested). This is significant since although these therapies are aimed to eliminate solid tumors, the genomic landscape of the tumor stroma is more significant in acquiring response to genotoxic stress, most likely harboring a response for both compartments. One of the significant results based on our networks is that hot or cold spot gene expression levels in stroma of tumor may lead to a significant benefit to cancer patients undergoing radiation therapy, which is commonly used in HNSCC treatment. Further investigation is needed to identify the pathways associated with these genes harboring response to radiation. We have also looked at NCI60 cell lines to see whether the general expression profiles of genes can be replicated in a wide range of tumor cell types. Although HNSCC cell lines do not exist in the NCI60 cell line array, this broad range of cells would give a general idea of how variations in gene expression among these cells would correlate to radiation response. Interestingly, expression of the hot/cold spot genes in the stroma network (14 genes) were statistically significant (Additional File 1, Table S11; p-value = 0.026) when correlated with response to radiation therapy.

**Table 4:** Microarray profiles of radiation response in the NCI60 cell lines

Gene Group	Genes	Tested	Statistic Q	Expected Q	sd of Q	p-value
Epithelium Network (EN)	21	20	0.12173	0.68791	0.70898	0.82857

Stroma Network (SN)	32	27	8.9873	6.0567	3.4807	0.2

Intersection of EN & SN	14	13	0.14672	1.0085	1.0742	0.85714

Hot/Cold spots in SN	14	11	7.2921	5.187	6.0014	0.28571

Hot/Cold spots in EN	9	8	0.050848	0.096741	0.05459	0.77143

Genes within 250 K of Epithelium Markers	79	61	18.863	8.8517	9.2183	0.17143

Genes within 250 K of Stroma Markers	235	151	56.296	10.686	10.746	0.02857

The network genes identified for stroma and epithelium of HNSCC as well as genes within 250 kb of stroma- and epithelium-associated markers are tested via Global Test [29], a statistical test scoring for association of the expression profile of groups of genes for outcome (e.g. response to radiation treatment: resistant/sensitive). Overall seven sets of genes were tested, and only hot- and cold-spot-associated genes within the stroma network (red/blue colored nodes in Figure 2) was shown to be significant, i.e., expression profiles of stroma-associated genes at hot-/cold-spots are associated with increased sensitivity to radiation. mRNA expression profiles are acquired from GEO (GSE9714), a study reporting gene expression profiles of radiosensitive and radioresistant human head and neck tumor cell lines [30].

## Conclusions

The proposed framework establishes valuable foundations towards building tumor-specific signaling pathway networks, which in return will provide a more thorough understanding of the pathobiology of HNSCC. The framework not only reduces the search space but also enables us to focus on specific proteins and genes that are active in HNSCC, including novel proteins related to molecular mechanisms involved in HNSCC. Pathways and networks are built up efficiently, utilizing widely available high-throughput data and providing powerful discovery tools for research. Our present work also demonstrates that this approach and framework can be applied to the tumor microenvironment whose role in tumorigenesis, invasion, progression and response in therapy will only gain in prominence [22,23,48].

## Methods

### Hot and cold spots of LOH/AI in HNSCC

In our study, we analyzed HNSCC samples that were previously collected and genotyped in an earlier study [16]. The two compartments of the neoplastic tissue (epithelium and stroma) in 122 samples (Table 1) were isolated using LCM as previously described in [16]. LOH/AI markers used in this study have coverage of 7 to 29 markers per chromosome, i.e. about 9-cM intermarker distance. In total, 366 microsatellite markers were analyzed in both epithelium and stroma samples from the 122 patients (Overall 244 samples, 122 epithelium and 122 stroma samples of the 122 patients). In this earlier study, all significant regions were named hot spots, attributing to their importance, regardless of their LOH/AI frequency.

In this study, *hot and cold spots of regional LOH/AI *are defined and identified. The LOH/AI regions that have significantly higher frequency of LOH/AI (*p*-value < 0.05) at a marker or markers compared with other markers along the same chromosome are named hot spots. In contrast if LOH/AI regions have significantly lower frequency of LOH/AI (*p*-value < 0.05) at a marker or markers compared with other markers along the same chromosome, we named these regions cold spots (See Table 2 for a summary, Additional File 1, Table S1 for epithelium hot/cold spots, and Additional File 1, Table S2 for stroma hot/cold spots). Since hot spot regions have significantly higher frequencies of LOH/AI, we expected to observe variation in these regions, whereas cold spots are regions that were less likely to carry variation. Among the 75 genomic locations of interest, 34 of them are *cold spots*, and 37 are *hot spots*. We also included four more markers that correlate to tumor size and nodal status that is not characterized as hot or cold spot in HNSCC (three of the four observed in stroma, and one in epithelium) [16].

### Tissue specific mRNA microarray data acquisition

Based on the assumption that genes affected by carcinogenesis should reflect their altered state on their respective mRNA expression levels, we acquired HNSCC somatic expression array data. Genes within close proximity of significant markers of LOH/AI can be associated with the available expression array data to further reveal relationships that can lead to clues about the role of these genes in biological pathways. Hence, publicly available microarray expression data for HNSCC is acquired from the Gene Expression Omnibus [32]. The array source is screened by the platform used, tissue (for controls) and tumors analyzed. The probe sets in this study are processed using the robust multiarray averaging (RMA). In this publicly available data set human gene expression levels were measured using Affymetrix U133 Plus 2.0 arrays. This array is a comprehensive genome-wide expression analysis chip that analyzes the expression level of over 47,000 transcripts and variants. We have merged all of the expression profiles and calculated the Pearson's Correlation Coefficient of all the genes over all the samples.

### Identifying signaling pathway networks of HNSCC

Network analysis frameworks are commonly used for computational analysis of high-throughput molecular interaction data and are useful in determining the conservation and divergence of functional organization in biological systems. Current widely used approaches are generally limited to specific target patterns, such as conserved sub-networks and motifs utilizing shortest path algorithms, nearest neighbor queries or topological properties based on limited abstractions from well annotated pathway databases such as KEGG. On the other hand, the computational framework utilized in this study (Figure 1) facilitates identification of components and features of the cellular network that characterize similarities and differences between cancerous and normal cells from a functional perspective. Details of this framework are given in the supplementary text.

### Identifying genes within vicinity of LOH/AI markers

First, possible LOH/AI regions of HNSCC are identified using a similar approach presented in [14]. Although carcinogenesis pathway of HNSCC does not directly represent functional relationships of cancer related genes, this information can be utilized to identify signaling pathways via a more systematic and integrated manner.

The LOH/AI regions identified from the signaling pathway will be associated with possible genes. Although each marker can correspond to more than one gene, by associating the genes with available high-throughput data that is related to cancer, genes can be eliminated. Here our assumption is that, if these LOH/AI regions are correlated with each other in terms of carcinogenesis, the effect should be observed in expression levels as well as interaction patterns of these genes after translation. Hence, if the genes within close proximity of these markers are associated with available high-throughput data, we should observe these relationships and eventually form hypotheses over functional relationships of these genes in terms of pathways. Previous studies [49-51] have used a similar approach, where they measured gene expression through mRNA levels to identify tumor suppressor genes in HNSCC. Identifying these hot spot genes will allow us to form a hypothesis of the functional relationships among these genes.

### Identifying gene expression correlation with outcome via Global Test

Global Test is a statistical test, giving a score for association of the expression profile of one or more groups of genes to a given outcome [29]. The test is based on the Cox proportional hazards model and is calculated using martingale residuals. This procedure allows us to test hypotheses about the influence of these groups of genes on survival directly; in our case response to genotoxic stress. A study reporting large-scale gene expression changes in response to genotoxic stress is utilized. The data is downloaded from GEO (accession GSE7505) [31]. In this dataset, radiation response was measured in NCI60 cell lines using NHGRI Homo sapiens 6 K array. Out of the 63 array samples, 15 cell lines were labeled as sensitive/resistant to genotoxic stress. Using these 15 samples and the seven set of genes (hot and cold spot genes (Additional File 1, Table S4 and Table S5), stroma and epithelium networks genes (Figure 2, Additional File 1, Table S6 and Table S7) and their intersection, and only the hot/cold spot genes that are identified in these two networks) the global test is run and p-values are acquired (Table 4). P-value calculation method was done using permutations over the whole microarray experiment downloaded. The number of permutations was limited to number of genes on each array used.

### Unspervised hierarchial clustering of gene expression profiles

The gene expression profile of each sample is first normalized by transforming values so that the mean is 0 and the standard deviation is 1. The clustering is performed by calculating Pearson's correlation coefficients between mRNA expression profiles over all samples and based on these distances building dendograms with hierarachical clustering method as it is implemented in Matlab (Matworks, Natick, MA). The heatmaps are generated based on the final clustering.

## Competing interests

The authors declare that they have no competing interests.

## Authors' contributions

CE conceptualized and directed the study. GB implemented the tools, and conducted the experiments. GB, MO and CE analyzed the data, interpreted the data and drafted the manuscript. All authors critically revised, reviewed and approved the final manuscript. CE had access to all the data and is responsible for the conduct and content of the study.

## Supplementary Material

Additional file 1 Supplementary Tables S1-S11 is provided in this file. Click here for file

Additional file 2 **Additional description of the methods followed in the study is provided in this file**. Click here for file

Additional file 3 Supplementary figures supporting the results is provided. Click here for file
