# Supplementary material for: Microenvironmental genomic alterations reveal signaling networks for head and neck squamous cell carcinoma
Source: J Clin Bioinforma. 2011 Aug 2;1:21. doi: 10.1186/2043-9113-1-21 (PMC3170587; doi:10.1186/2043-9113-1-21)
Supplement: Additional file 1 — Supplementary Tables S1-S11 is provided in this file. [file 2043-9113-1-21-S1.PDF]

Supplementary Tables

Microenvironmental Genomic Alterations Reveal Signaling Networks for Head and Neck Squamous Cell Carcinoma

Gurkan Bebek, Mohammed Orloff, Charis Eng

Table of contents:

Table S1: Significant markers identified from laser cut microdissected *epithelium* of HNSCC samples (Weber *et al.* 2007) are listed. The LOH/AI signal for each marker is compared with an offset for the same chromosome and p-values are calculated (fprp < 0.05). The Hot/Cold spot calls are made based on the signal-offset comparison. 2
Table S2: Significant markers identified from laser cut microdissected *stroma* of HNSCC samples (Weber *et al.* 2007) are listed. The LOH/AI signal for each marker is compared with an offset for the same chromosome and p-values are calculated (fprp < 0.05). The Hot/Cold spot calls are made based on the signal-offset comparison. 3
Table S3: The list of genes within 250Kb of the 75 DNA Markers investigated. The markers are annotated with their Hot/Cold spot status or whether they are associated with a clinicopathological feature (CPF). 5
Table S4: Table of Genes that are used for epithelium signaling pathway search, 21 Markers, 79 Genes 9
Table S5: Table of Genes that are used for stroma signaling pathway search, 48 markers 235 genes. 10
Table S6: The proto-oncogenes and tumor suppressor genes (TSG) identified in *HNSCC Stroma* are listed. The Hot/Cold spot definition is an identifier inherited from the DNA Marker the gene is associated with. The Compartment (Comp.) of each gene identifies the DNA Marker's compartment. 13
Table S7: The oncogenes and tumor suppressor genes identified in *HNSCC Epithelium* are listed. The Hot/Cold spot definition is an identifier inherited from the DNA Marker the gene is associated with. The Compartment (Comp.) of each gene identifies the DNA Marker's compartment. 16
Table S8: HNSCC LoH/AI Hot/Cold Spot spots that are also observed in structural varation studies. 18
Table S9: Methylation frequencies of genes associated with HNSCC are listed. The methylation degree shows the percentage of samples where methyltaion was detected in primary samples. Note that genes from the PubMeth Database that have no methylation in HNSCC are listed as well and shown with 0 at the bottom. (Ongenaert *et al.* 2008). 19
Table S10: HNSCC Positives: Genes that are well known to be associated with HNSCC are listed (Chin *et al.* 2004; Chen *et al.* 2008). 20
Table S11: Microarray profiles of radiation response in the NCI60 cell lines. 21
References: 22

Table S1: Significant markers identified from laser cut microdissected *epithelium* of HNSCC samples (Weber *et al.* 2007) are listed. The LOH/AI signal for each marker is compared with an offset for the same chromosome and p-values are calculated (fprp < 0.05). The Hot/Cold spot calls are made based on the signal-offset comparison.

Ept. Marker	Chr	Hot/Cold	p-value	power	fprp
D1S1596	1	Cold Spot	0.000263396	0.464756977	0.010653339
D1S518	1	Hot Spot	0.007017776	0.554507345	0.193848502
D1S1594	1	Cold Spot	0.008312359	0.387178697	0.289728326
D2S1394	2	Cold Spot	0.009755914	0.458277353	0.28799088
D3S4545	3	Hot Spot	0.007612002	0.601228734	0.193908601
D3S2403	3	Hot Spot	0.001261218	0.745175634	0.031155814
D3S1766	3	Hot Spot	0.014025475	0.575437394	0.316518879
D3S2460	3	Cold Spot	0.005276886	0.462499048	0.178159171
D5S1462	5	Cold Spot	0.014858664	0.384902613	0.423122499
D6S305	6	Hot Spot	0.024364372	0.46545847	0.498634506
D8S1128	8	Hot Spot	0.014521819	0.473649373	0.36810011
D10S1423	10	Hot Spot	0.019380934	0.445984778	0.452256888
D11S1999	11	Cold Spot	0.012661593	0.418122161	0.365223972
D12S2078	12	Hot Spot	0.001171866	0.535713498	0.039903766
D13S796	13	Cold Spot	0.011084278	0.544284031	0.278984478
D14S599	14	Cold Spot	8.42E-07	0.389814561	4.11E-05
D14S606	14	Hot Spot	0.002931402	0.426630451	0.115474832
D15S655	15	Hot Spot	0.004545436	0.456553132	0.159072894
D16S2616	16	Cold Spot	0.00924401	0.312315688	0.359945718
D16S422	16	Hot Spot	0.000208703	0.620957991	0.006345339
D17S2180	17	Cold Spot	0.005446391	0.444234431	0.188932686
GATA178F11	18	Hot Spot	0.023060282	0.440298759	0.498774311
D18S1376	18	Hot Spot	0.015356676	0.498710971	0.369109863
D19S591	19	Cold Spot	1.79E-05	0.531510173	0.000638213
D20S851	20	Cold Spot	0.006966367	0.436494572	0.232679402
D21S1437	21	Cold Spot	0.000625197	0.458277353	0.025265537
D21S2055	21	Hot Spot	0.003916045	0.656260262	0.101831687
D22S683	22	Hot Spot	0.017965645	0.55416328	0.381176143

Table S2: Significant markers identified from laser cut microdissected *stroma* of HNSCC samples (Weber *et al.* 2007) are listed. The LOH/AI signal for each marker is compared with an offset for the same chromosome and p-values are calculated (fprp < 0.05). The Hot/Cold spot calls are made based on the signal-offset comparison.

Stroma Marker	Chr	Hot/Cold	p-value	power	fprp
D1S3721	1	Hot Spot	0.004765944	0.545125747	0.1424508
D1S1596	1	Cold Spot	1.08E-07	0.514566487	3.97E-06
GATA133A08	1	Cold Spot	0.005611379	0.435600284	0.196630333
D2S1400	2	Cold Spot	0.000214194	0.469457172	0.008594417
D2S1394	2	Cold Spot	0.005729452	0.462499048	0.190527596
D2S1790	2	Cold Spot	0.00057186	1	0.010748551
D2S1334	2	Hot Spot	3.24E-05	0.521480066	0.001179501
D2S1776	2	Hot Spot	0.00808079	0.327099191	0.319442547
D3S2403	3	Hot Spot	0.012945049	0.333434237	0.424508288
D3S2432	3	Cold Spot	0.001051077	0.434521553	0.04394019
D3S2409	3	Hot Spot	0.026211674	0.524962875	0.486832125
D3S1766	3	Hot Spot	0.000472983	0.530913676	0.016645069
D3S1262	3	Cold Spot	0.017233431	0.410382216	0.443788914
D3S2418	3	Cold Spot	0.008878147	0.475776495	0.26174542
D4S1647	4	Cold Spot	0.001798896	0.440861668	0.071949687
D5S2500	5	Cold Spot	0.006777351	0.462395332	0.217823566
D5S1725	5	Cold Spot	0.001978938	0.375109065	0.091104932
D5S820	5	Hot Spot	0.012046329	0.481295329	0.322286854
D6S474	6	Cold Spot	0.000990946	0.277362866	0.063567054
D6S305	6	Hot Spot	0.021807147	0.434518129	0.488112005
D6S1027	6	Cold Spot	0.000786541	0.447730057	0.032299774
D7S3061	7	Cold Spot	0.001937135	0.398581551	0.084535256
D7S1804	7	Hot Spot	0.020741565	0.40116964	0.495548685
D7S3070	7	Cold Spot	0.002759306	0.500006858	0.094901546
D8S1477	8	Cold Spot	0.009896075	0.483562671	0.279971353
D10S1208	10	Hot Spot	0.002358375	0.522386128	0.079001247
D10S1230	10	Cold Spot	4.71E-05	0.381014032	0.002341264
D10S1222	10	Hot Spot	0.00633158	0.427102099	0.219765364
D11S4459	11	Hot Spot	0.002082408	0.51540966	0.071292803

Stroma Marker	Chr	Hot/Cold	p-value	power	fprp
D11S1998	11	Cold Spot	0.000371856	0.435600284	0.015960745
D11S4464	11	Hot Spot	0.011156344	0.452449695	0.319030828
D12S1042	12	Cold Spot	0.015026256	0.459876117	0.383027166
D12S297	12	Hot Spot	0.000947513	0.501892789	0.03462763
D12S1294	12	Hot Spot	0.014406499	0.481773165	0.362309319
D12S395	12	Hot Spot	0.001904719	0.511467302	0.066080903
D12S2078	12	Hot Spot	0.012884857	0.41512224	0.370964509
D13S787	13	Hot Spot	0.012880881	0.383806265	0.389371514
D13S285	13	Cold Spot	0.000925428	0.478032218	0.035477361
D14S1280	14	Hot Spot	0.012252252	0.363887942	0.390146324
D14S599	14	Cold Spot	5.29E-07	0.398956883	2.52E-05
D14S588	14	Cold Spot	0.015332697	0.463573177	0.385909921
D14S606	14	Hot Spot	0.000431966	0.48755178	0.016555128
D14S1434	14	Hot Spot	1.93E-06	0.691495292	5.31E-05
D16S403	16	Hot Spot	0.021038059	0.499837149	0.444353905
D16S3396	16	Cold Spot	0.002192282	0.330371295	0.111963989
D16S516	16	Hot Spot	0.013253284	0.38860796	0.393198608
D17S1308	17	Hot Spot	1.76E-06	0.756878015	4.42E-05
D17S1294	17	Hot Spot	0.004016306	0.427102099	0.151585242
D17S2180	17	Cold Spot	0.001355523	0.44942827	0.05420001
D19S591	19	Cold Spot	4.81E-06	0.541498716	0.000168734
D19S714	19	Hot Spot	0.003586532	0.532282266	0.113492873
D20S103	20	Cold Spot	0.000502288	0.339551028	0.027337802
D21S1437	21	Cold Spot	8.03E-05	0.489763452	0.003106844
D21S2052	21	Hot Spot	0.000305	0.587028696	0.009775236

Table S3: The list of genes within 250Kb of the 75 DNA Markers investigated. The markers are annotated with their Hot/Cold spot status or whether they are associated with a clinicopathological feature (CPF).

#	Marker ID	Location	Hot/Cold /CPF	*Genename*	Uniprot ID
1	D1S1596	1p32.1	Cold Spot	*FGGY*	Q96C11
2	D1S3721	1p34.2	Hot Spot	*CTPS*	P17812
3	D1S3721	1p34.2	Hot Spot	*EDN2*	P20800
4	D1S3721	1p34.2	Hot Spot	*HIVEP3*	Q9HCL7
5	D1S3721	1p34.2	Hot Spot	*SCMH1*	Q96GD3
6	D1S3721	1p34.2	Hot Spot	*SLFNL1*	Q499Z3
7	D1S1594	1q43	Cold Spot	*FMN2*	Q9NZ56
8	D1S1594	1q43	Cold Spot	*GREM2*	Q9H772
9	D1S1594	1q43	Cold Spot	*RGS7*	P49802
10	D2S1790	2p11.2	Cold Spot	*KCMF1*	Q9BWK2
11	D2S1790	2p11.2	Cold Spot	*LOC129293*	Q86V40
12	D2S1790	2p11.2	Cold Spot	*TMSB10*	P63313
13	D2S1394	2p13.2	Cold Spot	*EMX1*	Q04741
14	D2S1394	2p13.2	Cold Spot	*EXOC6B*	Q9Y2D4
15	D2S1394	2p13.2	Cold Spot	*RAB11FIP5*	Q9BXF6
16	D2S1394	2p13.2	Cold Spot	*SFXN5*	Q8TD22
17	D2S1394	2p13.2	Cold Spot	*SPR*	P35270
18	D2S1776	2p24.3	Hot Spot	*ABCB11*	O95342
19	D2S1776	2p24.3	Hot Spot	*G6PC2*	Q9NQR9
20	D2S1776	2p24.3	Hot Spot	*LASS6*	Q6ZMG9
21	D2S1776	2p24.3	Hot Spot	*NOSTRIN*	Q8IVI9
22	D2S1776	2p24.3	Hot Spot	*SPC25*	Q9HBM1
23	D2S1400	2p25.1	Cold Spot	*E2F6*	O75461
24	D2S1400	2p25.1	Cold Spot	*GREB1*	Q4ZG55
25	D2S1400	2p25.1	Cold Spot	*NTSR2*	O95665
26	D2S1400	2p25.1	Cold Spot	*ROCK2*	O75116
27	D2S1334	2q21.3	Hot Spot	*DARS*	P14868
28	D2S1334	2q21.3	Hot Spot	*LCT*	P09848
29	D2S1334	2q21.3	Hot Spot	*MCM6*	Q14566
30	D2S1334	2q21.3	Hot Spot	*R3HDM1*	Q15032
31	D2S1334	2q21.3	Hot Spot	*UBXD2*	Q92575
32	D2S1334	2q21.3	Hot Spot	*ZRANB3*	Q5FWF4
33	D3S1766	3p14.2	Hot Spot	*C3ORF67*	Q96C11
34	D3S2409	3p21.32	Hot Spot	*AMT*	P48728
35	D3S2409	3p21.32	Hot Spot	*BSN*	Q9UPA5
36	D3S2409	3p21.32	Hot Spot	*C3ORF62*	Q6ZUJ4
37	D3S2409	3p21.32	Hot Spot	*CCDC36*	Q8IYA8
38	D3S2409	3p21.32	Hot Spot	*CCDC71*	Q8IV32
39	D3S2409	3p21.32	Hot Spot	*DAG1*	Q14118
40	D3S2409	3p21.32	Hot Spot	*GPX1*	P07203
41	D3S2409	3p21.32	Hot Spot	*KLHDC8B*	Q8IXV7
42	D3S2409	3p21.32	Hot Spot	*LAMB2*	P55268
43	D3S2409	3p21.32	Hot Spot	*LOC646498*	NULL
44	D3S2409	3p21.32	Hot Spot	*NICN1*	Q9BSH3
45	D3S2409	3p21.32	Hot Spot	*RHOA*	P61586
46	D3S2409	3p21.32	Hot Spot	*TCTA*	P57738
47	D3S2409	3p21.32	Hot Spot	*USP4*	Q13107
48	D3S2432	3p22.3	Cold Spot	*CMTM8*	Q8IZV2
49	D3S2432	3p22.3	Cold Spot	*GPD1L*	Q14702
50	D3S2432	3p22.3	Cold Spot	*OSBPL10*	Q9BXB5
51	D3S4545	3p25.2	Hot Spot	*C3ORF32*	Q9Y2M2
52	D3S4545	3p25.2	Hot Spot	*CAV3*	P56539
53	D3S2403	3p25.2	Hot Spot	*IQSEC1*	Q6DN90
54	D3S4545	3p25.2	Hot Spot	*LMCD1*	Q9NZU5
55	D3S2403	3p25.2	Hot Spot	*NUP210*	Q8TEM1
56	D3S4545	3p25.2	Hot Spot	*OXTR*	P30559
57	D3S3630	3p26.3	CPF	*CNTN4*	Q8IWV2
58	D3S1262	3q27.2	Cold Spot	*AHSG*	P02765
59	D3S1262	3q27.2	Cold Spot	*CRYGS*	P22914
60	D3S1262	3q27.2	Cold Spot	*DGKG*	P49619
61	D3S1262	3q27.2	Cold Spot	*DNAJB11*	Q9UBS4
62	D3S1262	3q27.2	Cold Spot	*FETUB*	Q9UGM5
63	D3S1262	3q27.2	Cold Spot	*HRG*	P04196
64	D3S1262	3q27.2	Cold Spot	*KNG1*	P01042
65	D3S1262	3q27.2	Cold Spot	*TBCCD1*	Q9NVR7
66	D3S2418	3q28	Cold Spot	*C3ORF59*	Q8IYB1

#	Marker ID	Location	Hot/Cold /CPF	*Genename*	Uniprot ID
67	D3S2418	3q28	Cold Spot	*FGF12*	P61328
68	D4S1647	4q23	Cold Spot	*RAP1GDS1*	P52306
69	D4S1647	4q23	Cold Spot	*TSPAN5*	P62079
70	D5S2500	5q11.2	Cold Spot	*PDE4D*	Q08499
71	D5S1462	5q15	Cold Spot	*ERAP1*	Q9NZ08
72	D5S1462	5q15	Cold Spot	*ERAP2*	Q0V8I2
73	D5S1462	5q15	Cold Spot	*LIX1*	Q8N485
74	D5S1462	5q15	Cold Spot	*LNPEP*	Q9UIQ6
75	D5S1462	5q15	Cold Spot	*RIOK2*	Q9BVS4
76	D5S820	5q33.3	Hot Spot	*SGCD*	Q92629
77	D5S820	5q33.3	Hot Spot	*TIMD4*	Q96H15
78	D6S474	6q21	Cold Spot	*RFPL4B*	Q6ZWI9
79	D6S305	6q26	Hot Spot	*PARK2*	O60260
80	D6S1027	6q27	Cold Spot	*SMOC2*	Q9H3U7
81	D7S3061	7q31.32	Cold Spot	*ASB15*	Q8WXK1
82	D7S3061	7q31.32	Cold Spot	*HYAL4*	Q2M3T9
83	D7S3061	7q31.32	Cold Spot	*IQUB*	Q8NA54
84	D7S3061	7q31.32	Cold Spot	*LMOD2*	Q6P5Q4
85	D7S3061	7q31.32	Cold Spot	*NDUFA5*	Q16718
86	D7S3061	7q31.32	Cold Spot	*WASL*	O00401
87	D7S1804	7q32.3	Hot Spot	*CHCHD3*	Q9NX63
88	D7S1804	7q32.3	Hot Spot	*PLXNA4*	Q6BEA0
89	D7S3070	7q36.1	Cold Spot	*GALNT11*	Q8NCW6
90	D7S3070	7q36.1	Cold Spot	*GALNTL5*	Q7Z4T8
91	D7S3070	7q36.1	Cold Spot	*PRKAG2*	Q9UGJ0
92	D8S1477	8p12	Cold Spot	*NRG1*	Q02297
93	D8S1128	8q24.21	Hot Spot	*MYC*	P01106
94	D10S1208	10p11.21	Hot Spot	*CREM*	Q03060
95	D10S1208	10p11.21	Hot Spot	*CUL2*	Q13617
96	D10S1208	10p11.21	Hot Spot	*PARD3*	Q8TEW0
97	D10S1230	10q26.12	Cold Spot	*BRWD2*	Q9BZH6
98	D11S1999	11p15.4	Cold Spot	*AMPD3*	Q01432
99	D11S1999	11p15.4	Cold Spot	*CTR9*	Q6PD62
100	D11S1999	11p15.4	Cold Spot	*EIF4G2*	P78344
101	D11S1999	11p15.4	Cold Spot	*LYVE1*	Q9Y5Y7
102	D11S1999	11p15.4	Cold Spot	*MRVI1*	Q9Y6F6
103	D11S1999	11p15.4	Cold Spot	*RNF141*	Q8WVD5
104	D11S4459	11q12.1	Hot Spot	*OR5AK2*	Q8NH90
105	D11S4459	11q12.1	Hot Spot	*OR5AP2*	Q8NGF4
106	D11S4459	11q12.1	Hot Spot	*OR5AR1*	Q8NGP9
107	D11S4459	11q12.1	Hot Spot	*OR5M1*	Q8NGP8
108	D11S4459	11q12.1	Hot Spot	*OR5M10*	Q6IEU7
109	D11S4459	11q12.1	Hot Spot	*OR5M11*	Q96RB7
110	D11S4459	11q12.1	Hot Spot	*OR8U8*	P0C7N1
111	D11S4459	11q12.1	Hot Spot	*OR9G1*	Q8NH87
112	D11S4459	11q12.1	Hot Spot	*OR9G4*	Q8NGQ1
113	D11S4459	11q12.1	Hot Spot	*OR9G9*	P0C7N8
114	D11S1998	11q23.3	Cold Spot	*DSCAML1*	Q8TD84
115	D11S1998	11q23.3	Cold Spot	*FXYD2*	P54710
116	D11S1998	11q23.3	Cold Spot	*FXYD6*	Q9H0Q3
117	D11S1998	11q23.3	Cold Spot	*IL10RA*	Q13651
118	D11S1998	11q23.3	Cold Spot	*TMPRSS13*	Q9BYE2
119	D11S1998	11q23.3	Cold Spot	*TMPRSS4*	Q9NRS4
120	D11S4464	11q24.1	Hot Spot	*GRAMD1B*	Q9ULL9
121	D11S4464	11q24.1	Hot Spot	*OR10S1*	Q8NGN2
122	D11S4464	11q24.1	Hot Spot	*OR4D5*	Q8NGN0
123	D11S4464	11q24.1	Hot Spot	*OR6M1*	Q8NGM8
124	D11S4464	11q24.1	Hot Spot	*OR6T1*	Q8NGN1
125	D11S4464	11q24.1	Hot Spot	*OR6X1*	Q8NH79
126	D11S4464	11q24.1	Hot Spot	*OR8D4*	Q8NGM9
127	D11S4464	11q24.1	Hot Spot	*PMP22CD*	Q6GV28
128	D11S4464	11q24.1	Hot Spot	*SCN3B*	Q9NY72
129	D11S4464	11q24.1	Hot Spot	*ZNF202*	O95125
130	D12S1042	12p11.23	Cold Spot	*ARNTL2*	Q8WYA1
131	D12S1042	12p11.23	Cold Spot	*MRPS35*	P82673
132	D12S1042	12p11.23	Cold Spot	*PPFIBP1*	Q86W92
133	D12S1042	12p11.23	Cold Spot	*REP15*	Q6BDI9
134	D12S1042	12p11.23	Cold Spot	*STK38L*	Q9Y2H1
135	D12S297	12q13.13	Hot Spot	*ACVR1B*	P36896
136	D12S297	12q13.13	Hot Spot	*C12ORF44*	Q9BSB4
137	D12S297	12q13.13	Hot Spot	*GRASP*	Q7Z6J2
138	D12S297	12q13.13	Hot Spot	*KRT6B*	P04259
139	D12S297	12q13.13	Hot Spot	*KRT6C*	P48668
140	D12S297	12q13.13	Hot Spot	*KRT7*	P08729

#	Marker ID	Location	Hot/Cold /CPF	*Genename*	Uniprot ID
141	D12S297	12q13.13	Hot Spot	*KRT75*	O95678
142	D12S297	12q13.13	Hot Spot	*KRT80*	Q7Z3Q0
143	D12S297	12q13.13	Hot Spot	*KRT81*	Q14533
144	D12S297	12q13.13	Hot Spot	*KRT82*	Q9NSB4
145	D12S297	12q13.13	Hot Spot	*KRT83*	P78385
146	D12S297	12q13.13	Hot Spot	*KRT84*	Q9NSB2
147	D12S297	12q13.13	Hot Spot	*KRT85*	P78386
148	D12S297	12q13.13	Hot Spot	*KRT86*	O43790
149	D12S297	12q13.13	Hot Spot	*NR4A1*	P22736
150	D12S1294	12q21.33	Hot Spot	*CAND1*	Q86VP6
151	D12S1294	12q21.33	Hot Spot	*DYRK2*	Q92630
152	D12S395	12q24.23	Hot Spot	*CCDC60*	Q8IWA6
153	D12S395	12q24.23	Hot Spot	*CCDC64*	Q6ZP65
154	D12S395	12q24.23	Hot Spot	*CIT*	O14578
155	D12S395	12q24.23	Hot Spot	*PRKAB1*	Q9Y478
156	D13S787	13q12.12	Hot Spot	*MIPEP*	Q99797
157	D13S787	13q12.12	Hot Spot	*PCOTH*	Q58A44
158	D13S787	13q12.12	Hot Spot	*RP11-45B20.2*	B2RNN3
159	D13S787	13q12.12	Hot Spot	*TNFRSF19*	Q9NS68
160	D13S796	13q33.3	Cold Spot	*LOC728215*	NULL
161	D13S285	13q34	Cold Spot	*C13ORF28*	Q96KW9
162	D13S285	13q34	Cold Spot	*SOX1*	O00570
163	D14S599	14q13.1	Cold Spot	*C14ORF147*	Q969W0
164	D14S599	14q13.1	Cold Spot	*EGLN3*	Q9H6Z9
165	D14S588	14q24.1	Cold Spot	*KIAA0247*	Q92537
166	D14S588	14q24.1	Cold Spot	*SFRS5*	Q13243
167	D14S588	14q24.1	Cold Spot	*SLC10A1*	Q14973
168	D14S588	14q24.1	Cold Spot	*SMOC1*	Q9H4F8
169	D14S1434	14q32.13	Hot Spot	*DICER1*	Q9UPY3
170	D14S1434	14q32.13	Hot Spot	*GSC*	P56915
171	D14S1434	14q32.13	Hot Spot	*SERPINA3*	P01011
172	D14S1434	14q32.13	Hot Spot	*SERPINA5*	P05154
173	D16S403	16p12.1	Hot Spot	*HS3ST2*	Q9Y278
174	D16S403	16p12.1	Hot Spot	*SCNN1G*	P51170
175	D16S403	16p12.1	Hot Spot	*USP31*	Q70CQ4
176	D16S2616	16p13.3	Cold Spot	*A2BP1*	Q9NWB1

#	Marker ID	Location	Hot/Cold /CPF	*Genename*	Uniprot ID
177	D16S3396	16q12.1	Cold Spot	*SALL1*	Q9NSC2
178	D16S516	16q23.1	Hot Spot	*WWOX*	Q9NZC7
179	D16S422	16q23.3	Hot Spot	*CDH13*	P55290
180	D17S1308	17p13.3	Hot Spot	*FAM57A*	Q8TBR7
181	D17S1308	17p13.3	Hot Spot	*GEMIN4*	P57678
182	D17S1308	17p13.3	Hot Spot	*GLOD4*	Q9HC38
183	D17S1308	17p13.3	Hot Spot	*NXN*	Q7L4C6
184	D17S1308	17p13.3	Hot Spot	*RNMTL1*	Q53GN1
185	D17S1308	17p13.3	Hot Spot	*VPS53*	Q5VIR6
186	D17S1294	17q11.2	Hot Spot	*BLMH*	Q13867
187	D17S1294	17q11.2	Hot Spot	*CCDC55*	Q9H0G5
188	D17S1294	17q11.2	Hot Spot	*EFCAB5*	Q6ZRM6
189	D17S1294	17q11.2	Hot Spot	*SLC6A4*	P31645
190	D17S1294	17q11.2	Hot Spot	*SSH2*	Q76I76
191	D17S2180	17q21.32	Cold Spot	*C17ORF92*	Q96KF2
192	D17S2180	17q21.32	Cold Spot	*CALCOCO2*	Q13137
193	D17S2180	17q21.32	Cold Spot	*HOXB1*	P14653
194	D17S2180	17q21.32	Cold Spot	*HOXB13*	Q92826
195	D17S2180	17q21.32	Cold Spot	*HOXB2*	P14652
196	D17S2180	17q21.32	Cold Spot	*HOXB3*	P14651
197	D17S2180	17q21.32	Cold Spot	*HOXB4*	P17483
198	D17S2180	17q21.32	Cold Spot	*HOXB5*	P09067
199	D17S2180	17q21.32	Cold Spot	*HOXB6*	P17509
200	D17S2180	17q21.32	Cold Spot	*HOXB7*	P09629
201	D17S2180	17q21.32	Cold Spot	*HOXB8*	P17481
202	D17S2180	17q21.32	Cold Spot	*HOXB9*	P17482
203	D17S2180	17q21.32	Cold Spot	*SKAP1*	Q86WV1
204	D17S2180	17q21.32	Cold Spot	*TTLL6*	Q8N841
205	D18S843	18p11.22	CPF	*KIAA0802*	Q9Y4B5
206	D18S843	18p11.22	CPF	*PTPRM*	P28827
207	D18S843	18p11.22	CPF	*RAB12*	Q6IQ22
208	D18S1376	18q11.2	Hot Spot	*EPB41L3*	Q9Y2J2
209	D18S1376	18q11.2	Hot Spot	*ZFP161*	O43829
210	D19S714	19p13.12	Hot Spot	*AKAP8*	O43823
211	D19S714	19p13.12	Hot Spot	*AKAP8L*	Q9ULX6
212	D19S714	19p13.12	Hot Spot	*CYP4F12*	Q9HCS2
213	D19S714	19p13.12	Hot Spot	*CYP4F22*	Q6NT55

#	Marker ID	Location	Hot/Cold /CPF	*Genename*	Uniprot ID
214	D19S714	19p13.12	Hot Spot	*CYP4F3*	Q08477
215	D19S714	19p13.12	Hot Spot	*CYP4F8*	P98187
216	D19S714	19p13.12	Hot Spot	*FLJ21438*	Q8N2T9
217	D19S714	19p13.12	Hot Spot	*OR10H1*	Q9Y4A9
218	D19S714	19p13.12	Hot Spot	*OR10H2*	O60403
219	D19S714	19p13.12	Hot Spot	*OR10H3*	O60404
220	D19S714	19p13.12	Hot Spot	*OR10H5*	Q8NGA6
221	D19S714	19p13.12	Hot Spot	*PGLYRP2*	Q96PD5
222	D19S714	19p13.12	Hot Spot	*WIZ*	O95785
223	D19S591	19p13.3	Cold Spot	*AES*	Q08117
224	D19S591	19p13.3	Cold Spot	*BRUNOL5*	Q8N6W0
225	D19S591	19p13.3	Cold Spot	*GNA11*	P29992
226	D19S591	19p13.3	Cold Spot	*GNA15*	P30679
227	D19S591	19p13.3	Cold Spot	*NCLN*	Q969V3
228	D19S591	19p13.3	Cold Spot	*S1PR4*	O95977
229	D19S591	19p13.3	Cold Spot	*TLE2*	Q04725
230	D19S591	19p13.3	Cold Spot	*TLE6*	Q9H808
231	D19S591	19p13.3	Cold Spot	*ZNF554*	Q86TJ5
232	D19S591	19p13.3	Cold Spot	*ZNF555*	Q8NEP9
233	D19S591	19p13.3	Cold Spot	*ZNF556*	Q9HAH1
234	D19S591	19p13.3	Cold Spot	*ZNF57*	Q68EA5
235	D19S591	19p13.3	Cold Spot	*ZNF77*	Q15935
236	D19S559	19q13.31	CPF	*APOC1*	P02654
237	D19S559	19q13.31	CPF	*APOC2*	P02655
238	D19S559	19q13.31	CPF	*APOC4*	P55056
239	D19S559	19q13.31	CPF	*APOE*	P02649
240	D19S559	19q13.31	CPF	*BCAM*	P50895
241	D19S559	19q13.31	CPF	*BCL3*	P20749
242	D19S559	19q13.31	CPF	*CBLC*	Q9ULV8
243	D19S559	19q13.31	CPF	*CEACAM16*	Q2WEN9
244	D19S559	19q13.31	CPF	*CEACAM19*	Q7Z692

#	Marker ID	Location	Hot/Cold /CPF	*Genename*	Uniprot ID
245	D19S559	19q13.31	CPF	*CLPTM1*	O96005
246	D19S559	19q13.31	CPF	*PVR*	P15151
247	D19S559	19q13.31	CPF	*PVRL2*	Q92692
248	D19S559	19q13.31	CPF	*RELB*	Q01201
249	D19S559	19q13.31	CPF	*SFRS16*	Q8N2M8
250	D19S559	19q13.31	CPF	*TOMM40*	O96008
251	D19S559	19q13.31	CPF	*ZNF342*	Q8WUU4
252	D20S851	20p12.2	Cold Spot	*PLCB1*	Q9NQ66
253	D20S851	20p12.2	Cold Spot	*PLCB4*	Q15147
254	D20S103	20p13	Cold Spot	*C20ORF54*	Q9NQ40
255	D20S103	20p13	Cold Spot	*CSNK2A1*	P68400
256	D20S103	20p13	Cold Spot	*NRSN2*	Q9GZP1
257	D20S103	20p13	Cold Spot	*RBCK1*	Q9BYM8
258	D20S103	20p13	Cold Spot	*SCRT2*	Q9NQ03
259	D20S103	20p13	Cold Spot	*SOX12*	O15370
260	D20S103	20p13	Cold Spot	*SRXN1*	Q9BYN0
261	D20S103	20p13	Cold Spot	*TBC1D20*	Q96BZ9
262	D20S103	20p13	Cold Spot	*TCF15*	Q12870
263	D20S103	20p13	Cold Spot	*TRIB3*	Q96RU7
264	D21S2055	21q22.2	Hot Spot	*B3GALT5*	Q9Y2C3
265	D21S2055	21q22.2	Hot Spot	*DSCAM*	O60469
266	D21S2055	21q22.2	Hot Spot	*IGSF5*	Q9NSI5
267	D21S2055	21q22.2	Hot Spot	*PCP4*	P48539
268	D22S683	22q12.3	Hot Spot	*APOL1*	O14791
269	D22S683	22q12.3	Hot Spot	*APOL2*	Q9BQE5
270	D22S683	22q12.3	Hot Spot	*APOL3*	O95236
271	D22S683	22q12.3	Hot Spot	*APOL4*	Q9BPW4
272	D22S683	22q12.3	Hot Spot	*MYH9*	P35579
273	D22S683	22q12.3	Hot Spot	*RBM9*	O43251

Table S4: Table of Genes that are used for epithelium signaling pathway search, 21 Markers, 79 Genes

#	Marker ID	Genename
1	D1S1594	FMN2
2		GREM2
3		RGS7
4	D1S1596	FGGY
5	D2S1394	EMX1
6		EXOC6B
7		RAB11FIP5
8		SFXN5
9		SPR
10	D3S1766	C3orf67
11	D3S2403	IQSEC1
12		NUP210
14	D3S4545	C3orf32
16		CAV3
15		LMCD1
13		OXTR
17	D5S1462	ERAP1
18		ERAP2
19		LIX1
20		LNPEP
21		RIOK2
22	D6S305	PARK2
23	D8S1128	MYC
24	D11S1999	AMPD3
25		CTR9
26		EIF4G2
27		LYVE1
28		MRVI1
29		RNF141
30	D13S796	LOC728215
31	D14S599	C14orf147
32		EGLN3
33	D16S2616	A2BP1
34	D16S422	CDH13
35	D17S2180	C17orf92
36		CALCOCO2
37		HOXB1
38		HOXB13
39		HOXB2
40		HOXB3
41		HOXB4
42	D17S2180	HOXB5
43		HOXB6
44		HOXB7
45		HOXB8
46		HOXB9
47		SKAP1
48		TTLL6
49	D18S1376	EPB41L3
50		ZFP161
51	D18S843	KIAA0802
52		PTPRM
53		RAB12
54	D19S591	AES
55		BRUNOL5
56		EDG6
57		GNA11
58		GNA15
59		NCLN
60		S1PR4
61		TLE2
62		TLE6
63		ZNF554
64		ZNF555
65		ZNF556
66		ZNF57
67		ZNF77
68	D20S851	PLCB1
69		PLCB4
70	D21S2055	B3GALT5
71		DSCAM
72		IGSF5
73		PCP4
74	D22S683	APOL1
75		APOL2
76		APOL3
77		APOL4
78		MYH9
79		RBM9

Table S5: Table of Genes that are used for stroma signaling pathway search, 48 markers 235 genes.

#	Marker ID	Genename
1	D1S1596	FGGY
2	D1S3721	CTPS
3		EDN2
4		HIVEP3
5		SCMH1
6		SLFNL1
7	D2S1790	KCMF1
8		LOC129293
9		TMSB10
10	D2S1394	EMX1
11		EXOC6B
12		RAB11FIP5
13		SFXN5
14		SPR
15	D2S1776	ABCB11
16		G6PC2
17		LASS6
18		NOSTRIN
19		SPC25
20	D2S1400	E2F6
21		GREB1
22		NTSR2
23		ROCK2
24	D2S1334	DARS
25		LCT
26		MCM6
27		R3HDM1
28		UBXD2
29		ZRANB3
30	D3S1766	C3orf67
31	D3S2409	AMT
32		BSN
33		C3orf62
34		CCDC36

#	Marker ID	Genename
35		CCDC71
36		DAG1
37		GPX1
38		KLHDC8B
39		LAMB2
40		LOC646498
41		NICN1
42		RHOA
43		TCTA
44		USP4
45	D3S2432	CMTM8
46		GPD1L
47		OSBPL10
48	D3S2403	IQSEC1
49		NUP210
50	D3S3630	CNTN4
51	D3S1262	AHSG
52		CRYGS
53		DGKG
54		DNAJB11
55		FETUB
56		HRG
57		KNG1
58		TBCCD1
59	D3S2418	C3orf59
60		FGF12
61	D4S1647	RAP1GDS1
62		TSPAN5
63	D5S2500	PDE4D
64	D5S820	SGCD
65		TIMD4
66	D6S474	RFPL4B
67	D6S305	PARK2
68	D6S1027	SMOC2

#	Marker ID	Genename
69	D7S3061	ASB15
70		HYAL4
71		IQUB
72		LMOD2
73		NDUFA5
74		WASL
75	D7S1804	CHCHD3
76		PLXNA4
77	D7S3070	GALNT11
78		GALNTL5
79		PRKAG2
80	D8S1477	NRG1
81	D10S1208	CREM
82		CUL2
83		PARD3
84	D10S1230	BRWD2
85	D11S4459	OR5AK2
86		OR5AP2
87		OR5AR1
88		OR5M1
89		OR5M10
90		OR5M11
91		OR8U8
92		OR9G1
93		OR9G4
94		OR9G9
95	D11S1998	DSCAML1
96		FXYD2
97		FXYD6
98		IL10RA
99		TMPRSS13
100		TMPRSS4
101	D11S4464	GRAMD1B
102		OR10S1

#	Marker ID	Genename
103		OR4D5
104		OR6M1
105		OR6T1
106		OR6X1
107		OR8D4
108		PMP22CD
109		SCN3B
110		ZNF202
111	D12S1042	ARNTL2
112		MRPS35
113		PPFIBP1
114		REP15
115		STK38L
116	D12S297	ACVR1B
117		C12orf44
118		GRASP
119		KRT6B
120		KRT6C
121		KRT7
122		KRT75
123		KRT80
124		KRT81
125		KRT82
126		KRT83
127		KRT84
128		KRT85
129		KRT86
130		NR4A1
131	D12S1294	CAND1
132		DYRK2
133	D12S395	CCDC60
134		CCDC64
135		CIT
136		PRKAB1
137	D13S787	MIPEP
138		PCOTH
139		RP11-45B20.2

#	Marker ID	Genename
140		TNFRSF19
141	D13S285	C13orf28
142		SOX1
143	D14S599	C14orf147
144		EGLN3
145	D14S588	KIAA0247
146		SFRS5
147		SLC10A1
148		SMOC1
149	D14S1434	DICER1
150		GSC
151		SERPINA3
152		SERPINA5
153	D16S403	HS3ST2
154		SCNN1G
155		USP31
156	D16S3396	SALL1
157	D16S516	WWOX
158	D17S1308	FAM57A
159		GEMIN4
160		GLOD4
161		NXN
162		RNMTL1
163		VPS53
164	D17S1294	BLMH
165		CCDC55
166		EFCAB5
167		SLC6A4
168		SSH2
169	D17S2180	C17orf92
170		CALCOCO2
171		HOXB1
172		HOXB13
173		HOXB2
174		HOXB3
175		HOXB4
176		HOXB5

#	Marker ID	Genename
177		HOXB6
178		HOXB7
179		HOXB8
180		HOXB9
181		SKAP1
182		TTLL6
183	D19S714	AKAP8
184		AKAP8L
185		CYP4F12
186		CYP4F22
187		CYP4F3
188		CYP4F8
189		FLJ21438
190		OR10H1
191		OR10H2
192		OR10H3
193		OR10H5
194		PGLYRP2
195		WIZ
196	D19S591	AES
197		BRUNOL5
198		EDG6
199		GNA11
200		GNA15
201		NCLN
202		S1PR4
203		TLE2
204		TLE6
205		ZNF554
206		ZNF555
207		ZNF556
208		ZNF57
209		ZNF77
210	D19S559	APOC1
211		APOC2
212		APOC4
213		APOE

#	Marker ID	Genename
214		BCAM
215		BCL3
216		CBLC
217		CEACAM16
218		CEACAM19
219		CLPTM1
220		PVR
221		PVRL2

#	Marker ID	Genename
222		RELB
223		SFRS16
224		TOMM40
225		ZNF342
226	D20S103	C20orf54
227		CSNK2A1
228		NRSN2
229		RBCK1

#	Marker ID	Genename
230		SCRT2
231		SOX12
232		SRXN1
233		TBC1D20
234		TCF15
235		TRIB3

Table S6: The proto-oncogenes and tumor suppressor genes (TSG) identified in *HNSCC Stroma* are listed. The Hot/Cold spot definition is an identifier inherited from the DNA Marker the gene is associated with. The Compartment (Comp.) of each gene identifies the DNA Marker's compartment.

Category	Gene	Location	Description	Hot/Cold/CPF	Comp.	Ref:
TSG	ACVR1B	12q13.13	Activin receptor type-1B; Serine/threonine-protein kinase receptor R2	Hot Spot	Stroma	(Su et al. 2001)
-	ADAM15	1q21.3-1q22	A disintegrin and metalloproteinase domain (ADAM)15. A type I transmembrane glycoprotein, important in diverse biologic processes such as cell adhesion and proteolytic shedding of cell surface receptors.	-		
-	BSN	3p21.31	Protein bassoon; Zinc finger protein 231	Hot Spot	Stroma	
serine/ threonine protein kinase	CAMK2A	5q33.1	Calcium/calmodulin-dependent protein kinase type II alpha chain;	-		
-	CBLC	19q13.31	Signal transduction protein CBL-C; SH3-binding protein CBL-C; Regulator of EGFR mediated signal transduction.	-		
overexpressed in human cancers	CDC2	10q21.2	Cell division control protein 2 homolog; Cyclin-dependent kinase 1	CPF	Stroma	(Liu *et al.* 2008)
-	CNTN4	3p26.3	Contactin-4; Brain-derived immunoglobulin superfamily protein 2	CPF	Stroma	(Manderson *et al.* 2009)
Overexpression in adrenocortical cancer	CREM	10p11.21	cAMP-responsive element modulator; Inducible cAMP early repressor; ICER	Hot Spot	Stroma	(Groussin et al. 2000)
Proto-oncogene	CRK	17p13.3	Proto-oncogene C-crk; p38	-		(Reichman et al. 1992)
TSG	DOK2	8p21.3	Docking protein 2; Downstream of tyrosine kinase 2	-		(Niki *et al.* 2004)

Category	Gene	Location	Description	Hot/Cold/CPF	Comp.	Ref:
Proto-oncogene	EGFR	7p11.2	Epidermal growth factor receptor; Receptor tyrosine-protein kinase ErbB-1; EGFR and its ligands are cell signaling molecules involved in diverse cellular functions, including cell proliferation, differentiation, motility, and survival, and in tissue development.	-		
-	EMX1	2p13.2	Homeobox protein EMX1	Cold Spot	Ept/Str	
-	GRIN2B	12p13.1	Glutamate [NMDA] receptor subunit epsilon-2	-		
promotes tumor metastasis	GSC	14q32.13	Homeobox protein goosecoid	Hot Spot	Stroma	(Hartwell *et al.* 2006)
protein serine kinase	*GSK3A*	19q13.2	Glycogen synthase kinase-3 alpha;	-		
-	HOXB1	17q21.32	Homeobox protein Hox-B1	Cold Spot	Ept/Str	
involved in cervical cancer	HOXB4	17q21.32	Homeobox protein Hox-B4	Cold Spot	Ept/Str	(Lopez et al. 2006)
-	KRT82	12q13.13	Keratin type II cuticular Hb2	Hot Spot	Stroma	
Proto-oncogene	LCK	1p35.1	Proto-oncogene tyrosine-protein kinase LCK;	-		
-	LCT	2q21.3	Lactase-phlorizin hydrolase; Lactase	Hot Spot	Stroma	
Up regulation in cancer	NTSR2	2p25.1	Neurotensin receptor type 2	Cold Spot	Stroma	(Myers *et al.* 2009)
TSG	PARK2	6q26	Parkinson juvenile disease protein 2	Hot Spot	Ept/Str	(Cesari *et al.* 2003; Denison *et al.* 2003; Picchio *et al.* 2004; Wang *et al.* 2004)
Role in metastasis	PLCG1	20q12	1-phosphatidylinositol-4,5-bisphosphate phosphodiesterase gamma-1	-		(Sala et al. 2008)
TSG	PTPN6	12p13.31	Tyrosine-protein phosphatase non-receptor type 6	-		(Liedtke *et al.* 1998)
overexpressed in neuro-ectodermal tumors	PVR	19q13.31	Poliovirus receptor; Nectin-like protein 5;	CPF	Stroma	

Category	Gene	Location	Description	Hot/Cold/CPF	Comp.	Ref:
enhanced expression levels	PVRL1	11q23.3	Poliovirus receptor-related protein 1; Herpes virus entry mediator C;	-		(Kuner *et al.* 2009)
Oncogenic activity when overexpressed	RHOA	3p21.31	Transforming protein RhoA; H12;	Hot Spot	Stroma	(Fritz et al. 1999; Zhao et al. 2009) (Faried et al. 2005)
HNSCC biomarker	SFN	1p36.11	14-3-3 protein sigma; Stratifin; Epithelial cell marker protein 1	-		(Matta *et al.* 2008)
-	SKAP1	17q21.32	Src kinase-associated phosphoprotein 1	Cold Spot	Ept/Str	
Proto-oncogene	SRC	20q11.23	Proto-oncogene tyrosine-protein kinase Src;	-		
TSG	STAT5A	17q21.2	Signal transducer and activator of transcription 5A	-		(Zhang et al. 2007)
-	TLE6	19p13.3	Transducin-like enhancer protein 6	Cold Spot	Ept/Str	

Table S7: The oncogenes and tumor suppressor genes identified in *HNSCC Epithelium* are listed. The Hot/Cold spot definition is an identifier inherited from the DNA Marker the gene is associated with. The Compartment (Comp.) of each gene identifies the DNA Marker's compartment.

**Category**	**Gene**	**Location**	**Description**	**Hot /Cold/ CPF**	**Comp.**	**Ref.**
increase in thyroid cancers	ADRBK1	11q13.1	Beta-adrenergic receptor kinase 1, GRK2	-		(Metaye *et al.* 2008)
HNC Biomarker	CAV3	3p25.3	Caveolin-3; M-caveolin	Hot Spot	Ept	(Fine *et al.* 2005)
highly elevated expression	*CCR4*	3p22.3	C-C chemokine receptor type 4	-		(Ishida *et al.* 2006)
inactivated in multiple cancers	DAB1	1p32.2	Disabled homolog 1	-		(McAvoy *et al.* 2008)
TSG	DOK2	8p21.3	Docking protein 2; Downstream of tyrosine kinase 2	-		(Niki *et al.* 2004)
-	DSCAM	21q22.2	Down syndrome cell adhesion molecule	Hot Spot	Ept	
Proto-oncogene	EGFR	7p11.2	Epidermal growth factor receptor	-		
-	EMX1	2p13.2	Homeobox protein EMX1	Cold Spot	Ept/Str	
-	GRIN2B	12p13.1	Glutamate [NMDA] receptor subunit epsilon-2	-		
-	HOXB1	17q21.32	Homeobox protein HoxB1	Cold Spot	Ept/Str	
Involved in cervical cancer	HOXB4	17q21.32	Homeobox protein HoxB4	Cold Spot	Ept/Str	(Lopez *et al.*)
Proto-oncogene	LCK	1p35.1	Proto-oncogene tyrosine-protein kinase LCK; T cell-specific protein-tyrosine kinase	-		
TSG	PARK2	6q26	Parkinson juvenile disease protein 2	Hot Spot	Ept/Str	(Cesari *et al.* 2003; Denison *et al.* 2003; Picchio *et al.* 2004; Wang *et al.* 2004)
Role in metastasis	PLCB1	20p12.3	1-phosphatidylinositol-4,5-bisphosphate phosphodiesterase beta-1	Cold Spot	Ept	(Cocco *et al.* 2005)
Role in metastasis	PLCG1	20q12	1-phosphatidylinositol-4,5-bisphosphate phosphodiesterase gamma-1	-		(Sala *et al.* 2008)

Category	Gene	Location	Description	Hot /Cold/ CPF	Comp.	Ref.
serine/ threonine kinases	PRKCG	19q13.41-42	Protein kinase C gamma type	-		(Martiny-Baron *et al.* 2007)
TSG	PTPN6	12p13.31	Tyrosine-protein phosphatase non-receptor type 6	-		(Liedtke *et al.* 1998)
-	SKAP1	17q21.32	Src kinase-associated phosphoprotein 1	Cold Spot	Ept/Str	
Proto-oncogene	SRC	20q11.23	Proto-oncogene tyrosine-protein kinase Src	-		
TSG	STAT5A	17q21.2	Signal transducer and activator of transcription 5A	-		(Zhang *et al.* 2007)
-	TLE6	19p13.3	Transducin-like enhancer protein 6	Cold Spot	Ept/Str	

Table S8: HNSCC LoH/AI Hot/Cold Spot spots that are also observed in structural varation studies.

#	Study	Summary of the study	Result	Marker Location Shared	500K Regions shared
1	Conrad (Conrad et al. 2006)	Deletions from SNP genotype analysis	935 deletions	1	7
2	Hinds (Hinds et al. 2006)	Deletions from haploid hybridization analysis	100 deletions	0	0
3	Iafrate (Iafrate et al. 2004)	BAC microarray analysis	236 putative CNP regions	0	5
4	Locke (Locke et al. 2006)	CNP in duplication-rich regions	243 CNP regions	1	2
5	McCarroll (McCarroll et al. 2006)	Deletions from SNP genotype analysis	540 deletions	0	5
6	Redon (Redon *et al.* 2006)	SNP and BAC microarray analysis of HapMap data	1,445 CNV regions	6	23
7	Sebat (Sebat *et al.* 2004)	Representational oligonucleotide microarray analysis	80 putative CNP regions	0	0
8	Sharp (Sharp *et al.* 2005)	BAC microarray analysis	140 putative CNP regions	0	1
9	Tuzun (Tuzun *et al.* 2005)	Fosmid mapping	297 Intermediary SV sites	0	2
10	Itsara (Itsara *et al.* 2009)	Large CNV and Hotspots of Human Genetic Disease from SNP data analysis	13843 CNV calls from ~2500 samples using SNP arrays	19	49

Table S9: Methylation frequencies of genes associated with HNSCC are listed. The methylation degree shows the percentage of samples where methyltaion was detected in primary samples. Note that genes from the PubMeth Database that have no methylation in HNSCC are listed as well and shown with 0 at the bottom. (Ongenaert *et al.* 2008).

Gene	Methylation Degree	HNSCC Samples tested for methylation
SOCS3	80-100%	94
CDKN2B	60-80%	104
RARB2		27
STAT1		16
RARB	40-60%	32
CCNA1		20
UCHL1		20
CDKN2A	20-40%	400
MGMT		173
DAPK1		112
MLH1		170
CDH1		112
G0S2		20
MT1G		20
BMP2		20
RASSF1	0-20%	136
TP73		32
SOCS1		99
NMU		20
MSH2		123
KLK10	0%	0
ERS1		0
FHIT		0
EGFR		0
CADM1		0
CDH13		0
DCC		0
TIMP3		0
APC		0

Table S10: HNSCC Positives: Genes that are well known to be associated with HNSCC are listed (Chin *et al.* 2004; Chen *et al.* 2008).

**Group**	**GENE**
growth factors or growth factor receptors	*HST1*
	FGF3
	EGFR
	ERBB2
	PDGFB
intracellular signal transducers	*RAS*
	RAF1
	STAT3
transcription factors	*MYC*
	FOS
	JUN
	MYB
cell cycle regulators	*CCND1*
apoptosis	*BCL2*
	BAX
oncogenes	*FGF3*
	FGF4
	CCND1
	MYC
	MYCN
	KRAS
	NRAS
TSG	*CDKN2A*
	CDKN2B
Interleukins	*IL8*
Angiogenesis	*VEGF*

Table S11: Microarray profiles of radiation response in the NCI60 cell lines.

The network genes identified for stroma and epithelium of HNSCC as well as genes within 250K of stroma and epithelium DNA microsatellite markers are tested via Global Test [26], a statistical test scoring for association of the expression profile of groups of genes for outcome (e.g. response to radiation treatment: resistant/sensitive). Overall seven sets of genes are tested, and only hot and cold spot associated genes within the stroma network (red/blue colored nodes of stroma network in Figure 2) is shown to be significant, i.e. gene expression profiles are associated with sensitive response to radiation. mRNA Expression profiles are acquired from GEO (GSE7505) a study reporting large-scale gene expression changes in response to genotoxic stress, in which NCI Anti-Cancer Drug Screen (NCI60) cell lines are measured using NHGRI Homo sapiens 6K array [27].

Gene Group	Genes	Tested	Statistic Q	Expected Q	sd of Q	p-value
Epithelium Network (EN)	21	21	12.504	14.954	5.6829	0.61958
Stroma Network (SN)	32	32	10.629	9.1574	2.7139	0.25051
Intersection of EN & SN	14	14	9.375	10.685	3.9668	0.59425
Hot/Cold spots in SN	14	14	14.819	7.8017	3.1896	0.02595
Hot/Cold spots in EN	9	9	17.821	14.247	8.1575	0.27071
Genes within 250K of Str. Markers	79	65	10.575	9.7868	2.6664	0.33225
Genes within 250K of Ept. Markers	235	190	8.2092	7.9115	1.5303	0.36006

References:

Cesari, R., E. S. Martin, et al. (2003). "Parkin, a gene implicated in autosomal recessive juvenile parkinsonism, is a candidate tumor suppressor gene on chromosome 6q25-q27." Proc Natl Acad Sci U S A **100**(10): 5956-5961.

Chen, Y. and C. Chen (2008). "DNA copy number variation and loss of heterozygosity in relation to recurrence of and survival from head and neck squamous cell carcinoma: A review." Head & Neck **30**(10): 1361-1383.

Chin, D., G. M. Boyle, et al. (2004). "Molecular introduction to head and neck cancer (HNSCC) carcinogenesis." Br J Plast Surg **57**(7): 595-602.

Cocco, L., L. Manzoli, et al. (2005). "Nuclear phospholipase C beta1, regulation of the cell cycle and progression of acute myeloid leukemia." Adv Enzyme Regul **45**: 126-135.

Conrad, D. F., T. D. Andrews, et al. (2006). "A high-resolution survey of deletion polymorphism in the human genome." Nat Genet **38**(1): 75-81.

Denison, S. R., F. Wang, et al. (2003). "Alterations in the common fragile site gene Parkin in ovarian and other cancers." Oncogene **22**(51): 8370-8378.

Faried, A., M. Nakajima, et al. (2005). "Correlation between RhoA overexpression and tumour progression in esophageal squamous cell carcinoma." Eur J Surg Oncol **31**(4): 410-414.

Fine, S. W., M. P. Lisanti, et al. (2005). "Caveolin-3 Is a Sensitive and Specific Marker for Rhabdomyosarcoma." Applied Immunohistochemistry & Molecular Morphology **13**(3): 231-236.

Fritz, G., I. Just, et al. (1999). "Rho GTPases are over-expressed in human tumors." Int J Cancer **81**(5): 682-687.

Groussin, L., J. F. Massias, et al. (2000). "Loss of expression of the ubiquitous transcription factor cAMP response element-binding protein (CREB) and compensatory overexpression of the activator CREMtau in the human adrenocortical cancer cell line H295R." J Clin Endocrinol Metab **85**(1): 345-354.

Hartwell, K. A., B. Muir, et al. (2006). "The Spemann organizer gene, Goosecoid, promotes tumor metastasis." Proc Natl Acad Sci U S A **103**(50): 18969-18974.

Hinds, D. A., A. P. Kloek, et al. (2006). "Common deletions and SNPs are in linkage disequilibrium in the human genome." Nat Genet **38**(1): 82-85.

Iafrate, A. J., L. Feuk, et al. (2004). "Detection of large-scale variation in the human genome." Nat Genet **36**(9): 949-951.

Ishida, T. and R. Ueda (2006). "CCR4 as a novel molecular target for immunotherapy of cancer." Cancer Science **97**(11): 1139-1146.

Itsara, A., G. M. Cooper, et al. (2009). "Population Analysis of Large Copy Number Variants and Hotspots of Human Genetic Disease." The American Journal of Human Genetics **84**(2).

Kuner, R., T. Muley, et al. (2009). "Global gene expression analysis reveals specific patterns of cell junctions in non-small cell lung cancer subtypes." Lung Cancer **63**(1): 32-38.

Liedtke, M., P. Pandey, et al. (1998). "Regulation of Bcr-Abl-induced SAP kinase activity and transformation by the SHPTP1 protein tyrosine phosphatase." Oncogene **17**(15): 1889-1892.

Liu, P., T. P. Kao, et al. (2008). "CDK1 promotes cell proliferation and survival via phosphorylation and inhibition of FOXO1 transcription factor." Oncogene **27**(34): 4733-4744.

Locke, D. P., A. J. Sharp, et al. (2006). "Linkage disequilibrium and heritability of copy-number polymorphisms within duplicated regions of the human genome." Am J Hum Genet **79**(2): 275-290.

Lopez, R., E. Garrido, et al. (2006). "HOXB homeobox gene expression in cervical carcinoma." Int J Gynecol Cancer **16**(1): 329-335.

Manderson, E. N., A. H. Birch, et al. (2009). "Molecular genetic analysis of a cell adhesion molecule with homology to L1CAM, contactin 6, and contactin 4 candidate chromosome 3p26pter tumor suppressor genes in ovarian cancer." Int J Gynecol Cancer **19**(4): 513-525.

Martiny-Baron, G. and D. Fabbro (2007). "Classical PKC isoforms in cancer." Pharmacological Research **55**(6): 477-486.

Matta, A., L. V. DeSouza, et al. (2008). "Prognostic significance of head-and-neck cancer biomarkers previously discovered and identified using iTRAQ-labeling and multidimensional liquid chromatography-tandem mass spectrometry." J Proteome Res **7**(5): 2078-2087.

McAvoy, S., Y. Zhu, et al. (2008). "Disabled-1 is a large common fragile site gene, inactivated in multiple cancers." Genes, Chromosomes and Cancer **47**(2): 165-174.

McCarroll, S. A., T. N. Hadnott, et al. (2006). "Common deletion polymorphisms in the human genome." Nat Genet **38**(1): 86-92.

Metaye, T., P. Levillain, et al. (2008). "Immunohistochemical detection, regulation and antiproliferative function of G-protein-coupled receptor kinase 2 in thyroid carcinomas." J Endocrinol **198**(1): 101-110.

Myers, R. M., J. W. Shearman, et al. (2009). "Cancer, Chemistry, and the Cell: Molecules that Interact with the Neurotensin Receptors." ACS Chemical Biology **4**(7): 503-525.

Niki, M., A. Di Cristofano, et al. (2004). "Role of Dok-1 and Dok-2 in leukemia suppression." J Exp Med **200**(12): 1689-1695.

Ongenaert, M., L. Van Neste, et al. (2008). "PubMeth: a cancer methylation database combining text-mining and expert annotation." Nucleic Acids Res **36**(Database issue): D842-846.

Picchio, M. C., E. S. Martin, et al. (2004). "Alterations of the tumor suppressor gene Parkin in non-small cell lung cancer." Clin Cancer Res **10**(8): 2720-2724.

Redon, R., S. Ishikawa, et al. (2006). "Global variation in copy number in the human genome." Nature **444**(7118): 444-454.

Reichman, C. T., B. J. Mayer, et al. (1992). "The product of the cellular crk gene consists primarily of SH2 and SH3 regions." Cell Growth Differ **3**(7): 451-460.

Sala, G., F. Dituri, et al. (2008). "Phospholipase Cgamma1 is required for metastasis development and progression." Cancer Res **68**(24): 10187-10196.

Sebat, J., B. Lakshmi, et al. (2004). "Large-scale copy number polymorphism in the human genome." Science **305**(5683): 525-528.

Sharp, A. J., D. P. Locke, et al. (2005). "Segmental duplications and copy-number variation in the human genome." Am J Hum Genet **77**(1): 78-88.

Su, G. H., R. Bansal, et al. (2001). "ACVR1B (ALK4, activin receptor type 1B) gene mutations in pancreatic carcinoma." Proc Natl Acad Sci U S A **98**(6): 3254-3257.

Tuzun, E., A. J. Sharp, et al. (2005). "Fine-scale structural variation of the human genome." Nat Genet **37**(7): 727-732.

Wang, F., S. Denison, et al. (2004). "Parkin gene alterations in hepatocellular carcinoma." Genes Chromosomes Cancer **40**(2): 85-96.

Weber, F., Y. Xu, et al. (2007). "Microenvironmental genomic alterations and clinicopathological behavior in head and neck squamous cell carcinoma." JAMA **297**(1538-3598 (Electronic)).

Zhang, Q., H. Y. Wang, et al. (2007). "STAT5A is epigenetically silenced by the tyrosine kinase NPM1-ALK and acts as a tumor suppressor by reciprocally inhibiting NPM1-ALK expression." Nat Med **13**(11): 1341-1348.

Zhao, X., L. Lu, et al. (2009). "Overexpression of RhoA induces preneoplastic transformation of primary mammary epithelial cells." Cancer Res **69**(2): 483-491.
