# Supplementary material for: Microenvironmental genomic alterations reveal signaling networks for head and neck squamous cell carcinoma
Source: J Clin Bioinforma. 2011 Aug 2;1:21. doi: 10.1186/2043-9113-1-21 (PMC3170587; doi:10.1186/2043-9113-1-21)
Supplement: Additional file 2 — Additional description of the methods followed in the study is provided in this file. [file 2043-9113-1-21-S2.PDF]

Supplementary Materials

Microenvironmental Genomic Alterations Reveal Signaling Networks for Head and Neck Squamous Cell Carcinoma

Gurkan Bebek[1,3,4], Mohammed Orloff[1,2], Charis Eng[1,2,4,5 §]

[1]Genomic Medicine Institute and [2]Taussig Cancer Institute, Cleveland Clinic, 9500 Euclid Avenue, Mailstop NE-50 Cleveland, OH 44195, USA [3]Case Center for Proteomics and Bioinformatics, [4]Case Comprehensive Cancer Center and [5]Department of Genetics, Case Western Reserve University, 10900 Euclid Ave. Cleveland OH 44106, USA

[§]Corresponding author

This file contains Supplementary Methods and Supplementary Figures Legends

Table of contents:

Supplementary Methods 2

Identifying signaling pathway networks: 2

Supplementary Figure Legends 6

Reference: 8

Supplementary Methods

Identifying signaling pathway networks:

In this study, hot spot-, cold spot- and clinicopathological feature-associated LOH/AI regions were used to identify genes/proteins within proximity of these regions (250Kb). We have utilized a signaling network identification framework to discover networks connecting these genes [1-3]. In this framework, the underlying characteristics of relevant known pathways were captured by mapping information from the pathway proteins onto known functional annotations from Gene Ontology Database [4]. Next, the identified set of target genes are used to predict likely signaling pathway segments via this data-mining framework. The model incorporates known cancer-specific signaling pathways, cancer progression pathways and high-throughput experimental results including gene expression and protein-protein interactions to improve the outcome.

The mentioned framework has a number of steps. First, a reliable meta-network of interactions is created. Protein-protein interactions (PPIs) play a central role in the execution of key biological functions of a cell. PPIs of an organism can be summarized in a network in which each node represents a protein and each (undirected) edge represents an interaction. A graph including all proteins in an organism and all possible interactions between these proteins is called the protein-protein interaction network of that organism. This network is a collection of physical and genetic interactions available through legacy databases [5, 6]. These datasets are collected from high-throughput yeast two-hybrid assays [7, 8] or affinity purification followed by mass spectrometry [9, 10] as well as various pathways studies.

High-throughput experiments have been widely used to uncover physical interactions between proteins. However, these experiments identify only a small fraction of the total PPIN with questionable reliability [11, 12]. In this work, reliability scores to each PPI are assigned by integrating microarray expression levels, network topology, and protein subcellular localization data with a logistic regression model. The logistic regression model incorporates

four sets of variables for each interaction [1]. We utilize: (i) the number of times an interaction between two proteins was observed [13, 14], (ii) the Pearson correlation of expression measurements for the corresponding genes [15], (iii) the proteins' mutual clustering coefficient [16], and (iv) the binary (0/1) protein subcellular localization data of interacting partners [17, 18].

Gene expression profiles are integrated into this framework to increase the accuracy of PPIs. If a PPI is also identified as a signaling pathway interaction, then the genes producing the associated proteins should follow a similar or contrasting (activation or inhibition) level of expression. Previously, similar approaches were used to discover signaling pathways from available gene expression profiles of various cancer types and *S. cereviseae* [19-21]. We have collected microarray expression data for the tissue type in question, i.e., HNSCC [15].

Integration of protein subcellular localization eliminates interactions among proteins that are not biologically significant. False positive PPIs, proteins that erroneously appear to coexist at the same location in a cell are discarded. In this study, we also attempt to recover false negative PPIs [22]. A protein family is a group of evolutionarily related proteins. Therefore, proteins that are in the same family should have similar interaction patterns. Protein families of the interacting proteins are utilized to infer highly possible connections among proteins. In short, a protein family is a group of evolutionarily related proteins. Therefore, by grouping the proteins, we capture their possible interaction traits and can infer highly possible missing interactions. Previously, interactions for *S. cereviseae* were inferred with an accuracy of 79% [22]. We grow the PPIN with these inferred interaction edges and carry out our searches on this extended network as well as the actual reported interactions.

Next, functional annotations of known pathway proteins are collected to capture underlying characteristics of these pathways. The characteristics are then utilized to search for possible (unknown) pathway segments. Biological annotations, e.g., Gene Ontology [4], provide a basis to find functionally similar proteins. Using functional annotations instead of

the protein itself would better capture the true relationships among proteins in known pathways. Association rule mining, a procedure to collect data attributes that are statistically related in the underlying data, is used to discover these patterns. By collecting the underlying patterns of signaling pathways, a library of templates is generated. These rules are then used to evaluate the candidate pathway segments for possible occurrences of these rules. By extracting associations of the proteins in pathways, associations of connected proteins are examined to see whether they interact with each other in a similar way or not.

Our goal is to discover possible signaling pathway segments connecting our candidate genes/proteins. We query our rule set for every pair of candidate genes/proteins. For each pair of targets, we check every path between these two nodes on the filtered interaction network. We check the annotations of the proteins on this segment and count the association rules. We further filter each path identified by their average value of expression profiles (average absolute Pearson coefficients ≥ 0.7) to make sure the genes on each path are correlated. Finally we return paths with significant interactions.

As shown in the connectivity arrays in Figure S1, the number of candidate genes introduced into the framework is significantly reduced via the computational data processing steps that associated high throughput data sets with these genes. In this array, genes from the same marker vicinity are grouped together. Connectivity arrays are a way of representing relationships of genes with other candidate genes with a significant signaling path of length l or less. Paths are then brought together to generate signaling pathway networks.

In this search process, we only allow one inferred edge (recovered false negative edge) where possible. Finally, all simple paths that are found to be significant are pooled together and the interaction edges that are unlikely to appear in high frequencies (edges with a significant p-value) are returned as the candidate pathway network.

LOH/AI gene identification

In this study, hot/cold spot marker locations (as defined in main text) are utilized to identify a set of genes for further pathway evaluation. Since the microsatellite markers are small in size, and are spread out on the genome, if genes that only overlap with these markers are used, the set of genes that we would utilize in this study would be limited to 27 genes. In order to make sure we include the regions that the markers are pointing out, we have extended these regions at variable lengths to identify genes within proximity of these markers. Shown in Figure S4, we have extended regions from 100 Kb to the borders of the loci that they reside in. The linear increase shows the increasing number of genes associated with marker locations. The computational methodology as described in [1, 2] and summarized above would increasingly take more computational time as the regions are extended even more. In this study, we have chosen 250 Kb for these extensions, where a large set of genes were included for analysis in a reasonable time.

Supplementary Figure Legends

Figure S1 - Connectivity array of the target genes

The matrices depict the pairwise connectivity of target genes in (A) epithelium and (B) stroma of HNSCC. The color in each cell represents one of the four possible outcomes of a pairwise search. *Black*: There is no path on the network connecting the two proteins. *Red*: There is at least one path on the network connecting the two proteins, but it does not carry significant coexpression correlation and annotations. *White*: There is a path with significant coexpression correlation and annotation. *Yellow*: There is a significant path including an estimated edge

Figure S2 - Random set of genes clustered

mRNA expression levels of a set of randomly picked genes are clustered using hierarchical clustering. Horizontal clustering depicts genes and vertical clustering group tissue samples [23] through expression levels. The labels below represent HNSCC tumors with HPV status (+ or -) and normal oral cavity and tonsil tissue samples included in the mRNA expression microarray study [15].

Figure S3 - Matched HNSCC HPV+/- samples clustered via mRNA expression levels of commonly hypermethylated genes in HNSCC

PubMeth (Reviewed methylation database of cancer genes, [24]) genes associated with HNSCC that are known to have some degree of hypermethylation are clustered using hierarchical clustering. The samples are matched by tissue type and stage. The clustering depicts separation of HPV+ and HPV- samples. Univariate analysis identified 10/19 genes as significant based on HPV+/- status (FDR corrected p-value <0.05).

Figure S4 – Number of genes within proximity of marker locations

Significant markers locations are extended at variable lengths to identify the number of genes within proximity of these markers. The extension varies from none to same loci. A linear increase in the number of genes has been observed.

Reference:

1. Bebek G, Yang J: **PathFinder: mining signal transduction pathway segments from protein-protein interaction networks.** *BMC Bioinformatics* 2007, **8**.

2. Bebek G, Patel V, Chance MR: **PETALS: Proteomic Evaluation and Topological Analysis of a mutated Locus' Signaling.** *BMC Bioinformatics* 2010, **11**:596.

3. Patel VN, Bebek G, Mariadason JM, Wang D, Augenlicht LH, Chance MR: **Prediction and testing of biological networks underlying intestinal cancer.** *PLoS One* 2010, **5**.

4. Ashburner M, Ball CA, Blake JA, Botstein D, Butler H, Cherry JM, Davis AP, Dolinski K, Dwight SS, Eppig JT, et al: **Gene ontology: tool for the unification of biology. The Gene Ontology Consortium.** *Nat Genet* 2000, **25**.

5. Mishra GR, Suresh M, Kumaran K, Kannabiran N, Suresh S, Bala P, Shivakumar K, Anuradha N, Reddy R, Raghavan TM, et al: **Human protein reference database--2006 update.** *Nucleic Acids Res* 2006, **34**.

6. Kerrien S, Alam-Faruque Y, Aranda B, Bancarz I, Bridge A, Derow C, Dimmer E, Feuermann M, Friedrichsen A, Huntley R, et al: **IntAct--open source resource for molecular interaction data.** *Nucleic Acids Res* 2007, **35**.

7. Fields S, Song O: **A novel genetic system to detect protein-protein interactions.** *Nature* 1989, **340**.

8. Walhout AJ, Vidal M: **High-throughput yeast two-hybrid assays for large-scale protein interaction mapping.** *Methods* 2001, **24**:297-306.

9. Gavin AC, others, Bsche M, Krause R, Grandi P, Marzioch M, Bauer A, Schultz J, Rick JM, Michon AM, et al: **Functional organization of the yeast proteome by systematic analysis of protein complexes.** *Nature* 2002, **415**.

10. Ho Y, others: **Systematic identification of protein complexes in Saccharomyces cerevisiae by mass spectrometry.** *Nature* 2002, **415**.

11. Grigoriev A: **On the number of protein-protein interactions in the yeast proteome.** *Nuc Ac Res* 2003, **31**.

12. von Mering C, Krause R, Snel B, Cornell M, Oliver SG, Fields S, Bork P: **Comparative assessment of large-scale data sets of protein-protein interactions.** *Nature* 2002, **417**.

13. Deng M, Sun F, Chen T: **Assessment of the reliability of protein-protein interactions and protein function prediction.** *Pac Symp Biocomput* 2003.

14. Sharan R, Suthram S, Kelley RM, Kuhn T, McCuine S, Uetz P, Sittler T, Karp RM, Ideker T: **Conserved patterns of protein interaction in multiple species.** *Proc Natl Acad Sci U S A* 2005, **102**.

15. Slebos RJ, Yi Y, Ely K, Carter J, Evjen A, Zhang X, Shyr Y, Murphy BM, Cmelak AJ, Burkey BB, et al: **Gene expression differences associated with human**

papillomavirus status in head and neck squamous cell carcinoma. *Clin Cancer Res* 2006, **12**.

16. Goldberg DS, Roth FP, Goldberg DS, Roth FP: **Assessing experimentally derived interactions in a small world.** *Proc Natl Acad Sci U S A* 2003, **100**.

17. Schneider M, Lane L, Boutet E, Lieberherr D, Tognolli M, Bougueleret L, Bairoch A: **The UniProtKB/Swiss-Prot knowledgebase and its Plant Proteome Annotation Program.** *J Proteomics* 2008.

18. Sprenger J, Lynn Fink J, Karunaratne S, Hanson K, Hamilton NA, Teasdale RD, Sprenger J, Karunaratne S, Hanson K, Hamilton NA, Teasdale RD: **LOCATE: a mammalian protein subcellular localization database.** *Nucleic Acids Res* 2008, **36**.

19. Steffen M, Petti A, Aach J, D`haeseleer P, Church G: **Automated modelling of signal transduction networks.** *BMC Bioinformatics* 2002, **3**.

20. Graeber TG, Eisenberg D: **Bioinformatic identification of potential autocrine signaling loops in cancers from gene expression profiles.** *Nat Genet* 2001, **29**.

21. Liu Y, Zhao H: **A computational approach for ordering signal transduction pathway components from genomics and proteomics Data.** *BMC Bioinformatics* 2004, **5**.

22. Bebek G, Takamoto K: **Accurate Elimination of False Protein-Protein Interactions.** In *RECOMB 2007*; *2007*. 2007

23. Pyeon D, Newton MA, Lambert PF, den Boon JA, Sengupta S, Marsit CJ, Woodworth CD, Connor JP, Haugen TH, Smith EM, et al: **Fundamental differences in cell cycle deregulation in human papillomavirus-positive and human papillomavirus-negative head/neck and cervical cancers.** *Cancer Res* 2007, **67:**4605-4619.

24. Ongenaert M, Van Neste L, De Meyer T, Menschaert G, Bekaert S, Van Criekinge W: **PubMeth: a cancer methylation database combining text-mining and expert annotation.** *Nucleic Acids Res* 2008, **36:**D842-846.

25. Chen Y, Chen C: **DNA copy number variation and loss of heterozygosity in relation to recurrence of and survival from head and neck squamous cell carcinoma: A review.** *Head & Neck* 2008, **30:**1361-1383.

26. Chin D, Boyle GM, Theile DR, Parsons PG, Coman WB: **Molecular introduction to head and neck cancer (HNSCC) carcinogenesis.** *Br J Plast Surg* 2004, **57:**595-602.

27. Chung CH, Parker JS, Karaca G, Wu J, Funkhouser WK, Moore D, Butterfoss D, Xiang D, Zanation A, Yin X, et al: **Molecular classification of head and neck squamous cell carcinomas using patterns of gene expression.** *Cancer Cell* 2004, **5**.
