# Supplementary material for: Microenvironmental genomic alterations reveal signaling networks for head and neck squamous cell carcinoma
Source: J Clin Bioinforma. 2011 Aug 2;1:21. doi: 10.1186/2043-9113-1-21 (PMC3170587; doi:10.1186/2043-9113-1-21)
Supplement: Additional file 3 — Supplementary figures supporting the results is provided. [file 2043-9113-1-21-S3.PDF]

Supplementary Figures

Microenvironmental Genomic Alterations Reveal Signaling Networks for Head and Neck Squamous Cell Carcinoma

Gurkan Bebek[1,3,4], Mohammed Orloff[1,2], Charis Eng[1,2,4,5 §]

[1]Genomic Medicine Institute and [2]Taussig Cancer Institute, Cleveland Clinic, 9500 Euclid Avenue, Mailstop NE-50 Cleveland, OH 44195, USA
[3]Case Center for Proteomics and Bioinformatics, [4]Case Comprehensive Cancer Center and [5]Department of Genetics, Case Western Reserve University, 10900 Euclid Ave. Cleveland OH 44106, USA

[§]Corresponding author

Figure S1 A

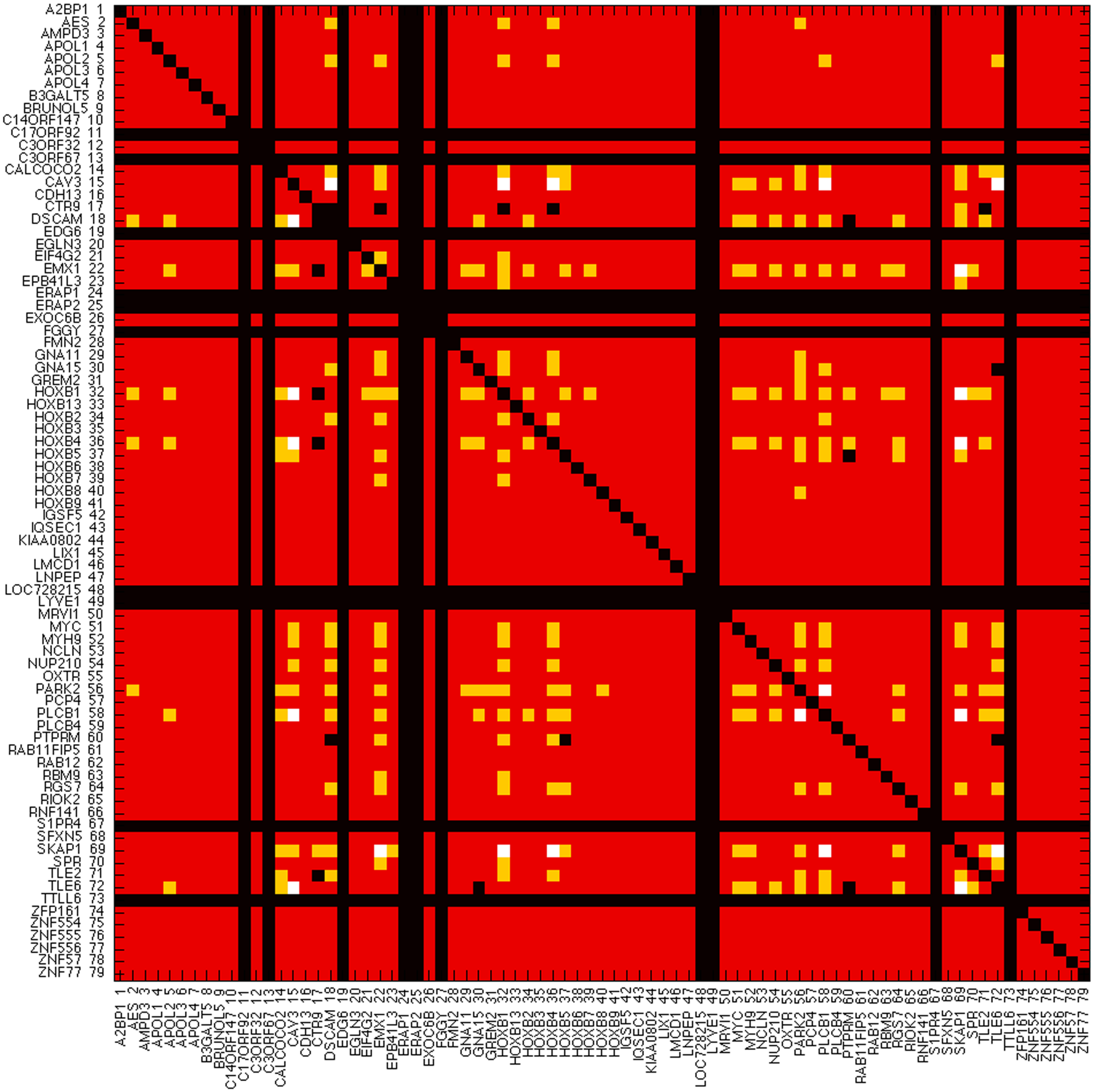
A2BP1 1
AES 2
AMPD3 3
APOL1 4
APOL2 5
APOL3 6
APOL4 7
B3GALT5 8
BRUNOL5 9
C14ORF147 10
C17ORF92 11
C3ORF32 12
C3ORF67 13
CALCOCO2 14
CAV3 15
CDH13 16
CTR9 17
DSCAM 18
EDG6 19
EGLN3 20
EIF4G2 21
EMX1 22
EPB41L3 23
ERAP1 24
ERAP2 25
EXOC6B 26
FGGY 27
FMN2 28
GNA11 29
GNA15 30
GREM2 31
HOXB1 32
HOXB13 33
HOXB2 34
HOXB3 35
HOXB4 36
HOXB5 37
HOXB6 38
HOXB7 39
HOXB8 40
HOXB9 41
IGSF5 42
IQSEC1 43
KIAA0802 44
LIX1 45
LMCD1 46
LNPEP 47
LOC728215 48
LYVE1 49
MRVI1 50
MYC 51
MYH9 52
NCLN 53
NUP210 54
OXTR 55
PARK2 56
PCP4 57
PLCB1 58
PLCB4 59
PTPRM 60
RAB11FIP5 61
RAB12 62
RBM9 63
RGS7 64
RIOK2 65
RNF141 66
S1PR4 67
SFXN5 68
SKAP1 69
SPR 70
TLE2 71
TLE6 72
TTLL6 73
ZFP161 74
ZNF554 75
ZNF555 76
ZNF556 77
ZNF57 78
ZNF77 79

Figure S1 B

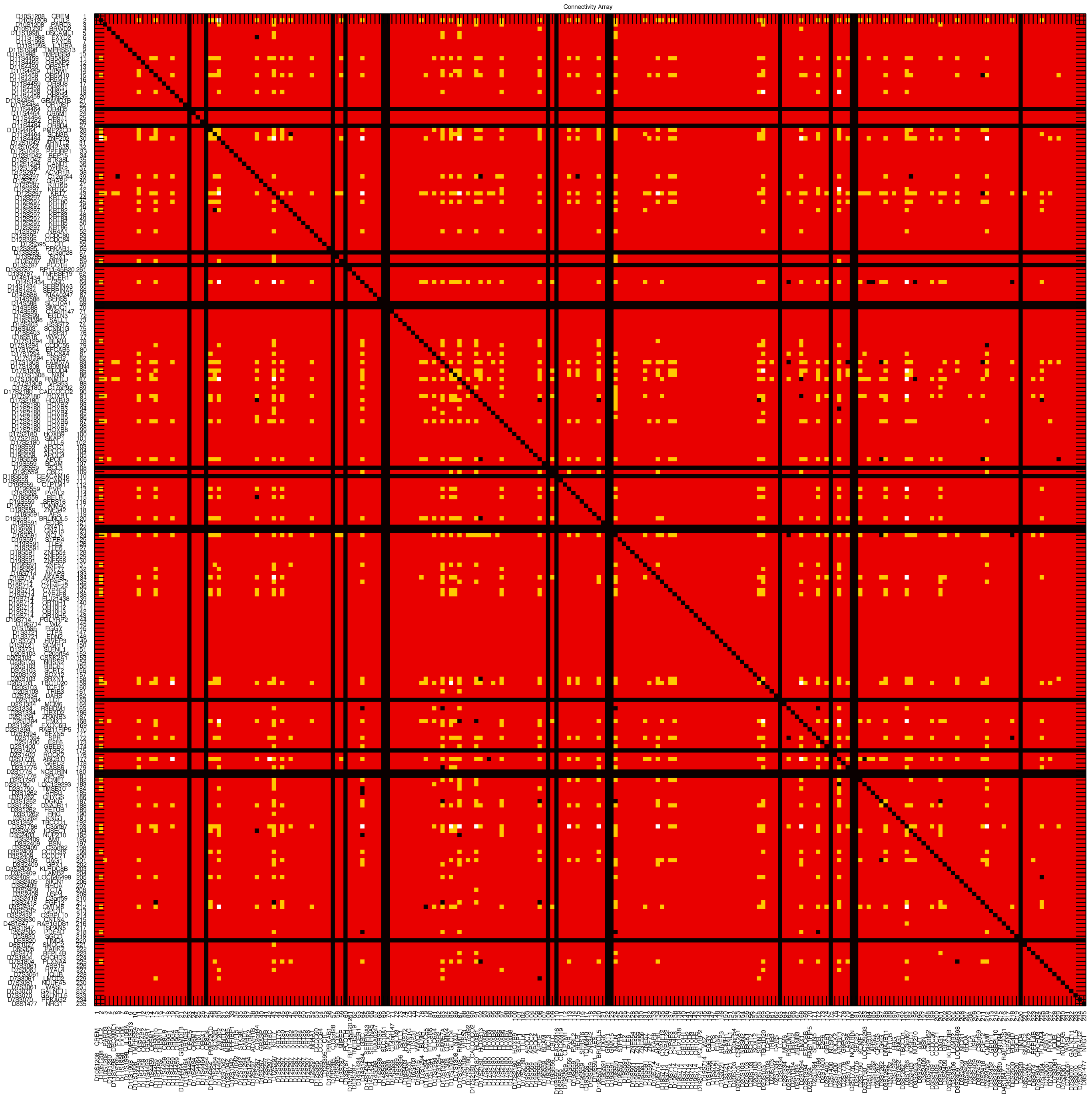

Figure S2

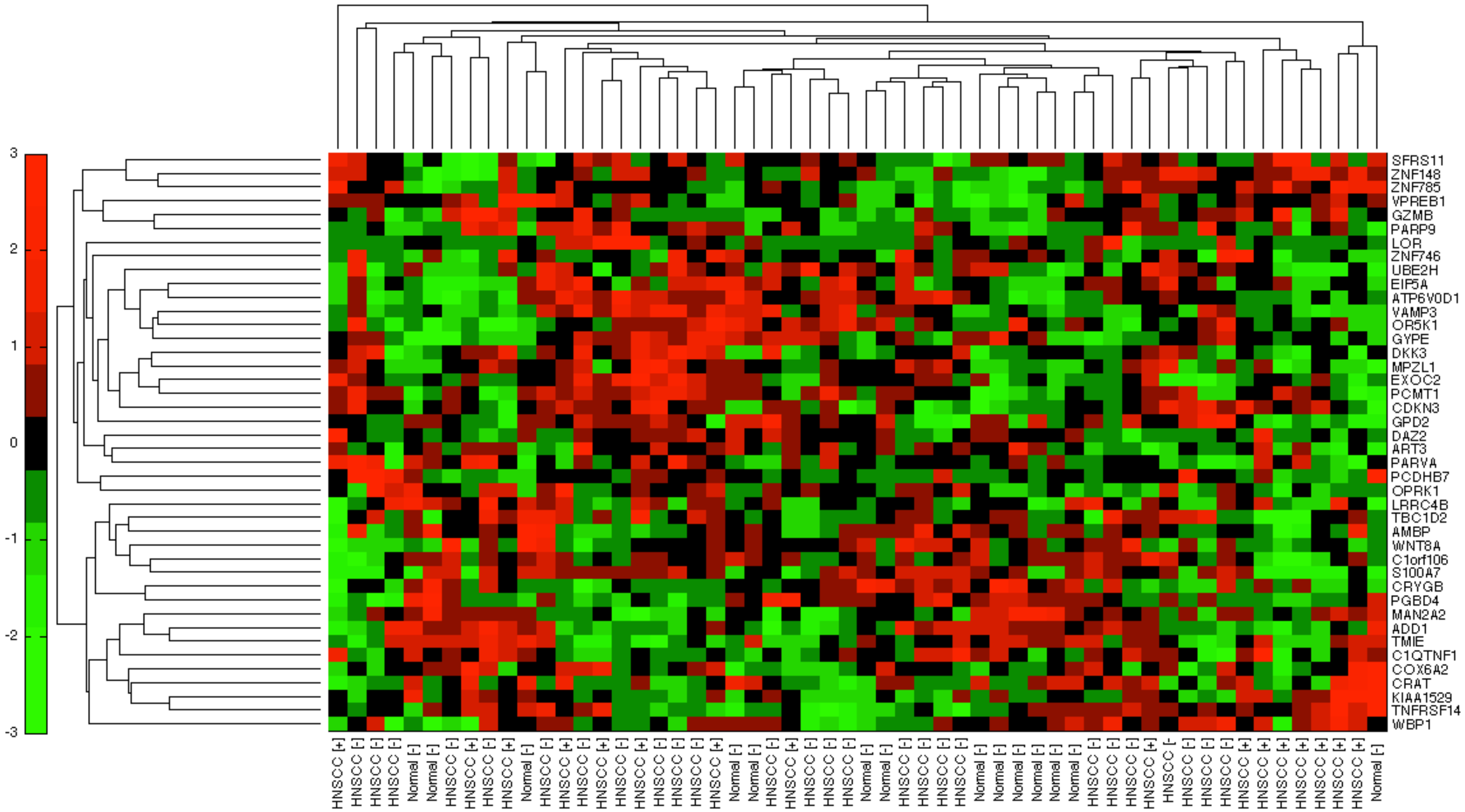
3
2
1
0
-1
-2
-3
SFRS11
ZNF148
ZNF785
VPREB1
GZMB
PARP9
LOR
ZNF746
UBE2H
EIF5A
ATP6V0D1
VAMP3
OR5K1
GYPE
DKK3
MPZL1
EXOC2
PCMT1
CDKN3
GPD2
DAZ2
ART3
PARVA
PCDHB7
OPRK1
LRRC4B
TBC1D2
AMBP
WNT8A
C1orf106
S100A7
CRYGB
PGBD4
MAN2A2
ADD1
TMIE
C1QTNF1
COX6A2
CRAT
KIAA1529
TNFRSF14
WBP1
HNSCC [+]
HNSCC [-]
HNSCC [-]
HNSCC [-]
Normal [-]
Normal [-]
HNSCC [-]
HNSCC [+]
HNSCC [-]
HNSCC [+]
Normal [-]
HNSCC [-]
HNSCC [+]
HNSCC [-]
HNSCC [+]
HNSCC [-]
HNSCC [+]
HNSCC [-]
HNSCC [-]
HNSCC [-]
HNSCC [+]
Normal [-]
Normal [-]
HNSCC [-]
HNSCC [+]
HNSCC [-]
HNSCC [-]
HNSCC [-]
Normal [-]
Normal [-]
HNSCC [-]
HNSCC [-]
HNSCC [-]
HNSCC [-]
Normal [-]
Normal [-]
Normal [-]
Normal [-]
Normal [-]
Normal [-]
HNSCC [-]
HNSCC [-]
HNSCC [-]
HNSCC [+]
HNSCC [-
HNSCC [-]
HNSCC [-]
HNSCC [-]
HNSCC [+]
HNSCC [+]
HNSCC [+]
HNSCC [+]
HNSCC [+]
HNSCC [+]
HNSCC [+]
Normal [-]

Figure S3

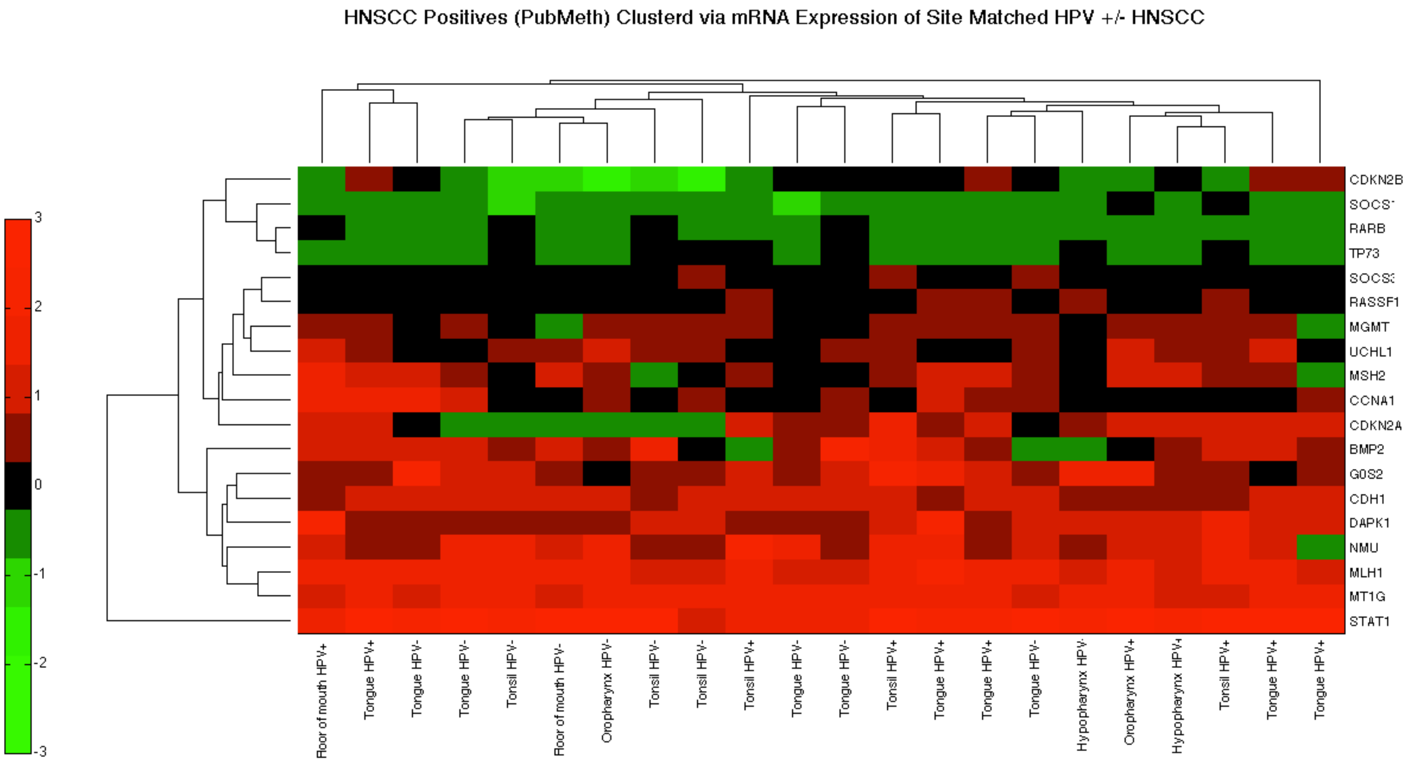

HNSCC Positives (PubMeth) Clusterd via mRNA Expression of Site Matched HPV +/- HNSCC
3
2
1
0
-1
-2
-3
CDKN2B
SOCS1
RARB
TP73
SOCS3
RASSF1
MGMT
UCHL1
MSH2
CCNA1
CDKN2A
BMP2
G0S2
CDH1
DAPK1
NMU
MLH1
MT1G
STAT1
Floor of mouth HPV+
Tongue HPV+
Tongue HPV-
Tongue HPV-
Tonsil HPV-
Floor of mouth HPV-
Oropharynx HPV-
Tonsil HPV-
Tonsil HPV-
Tonsil HPV+
Tongue HPV-
Tongue HPV-
Tonsil HPV+
Tongue HPV+
Tongue HPV+
Tongue HPV-
Hypopharynx HPV-
Oropharynx HPV+
Hypopharynx HPV+
Tonsil HPV+
Tongue HPV+
Tongue HPV+

Figure S4

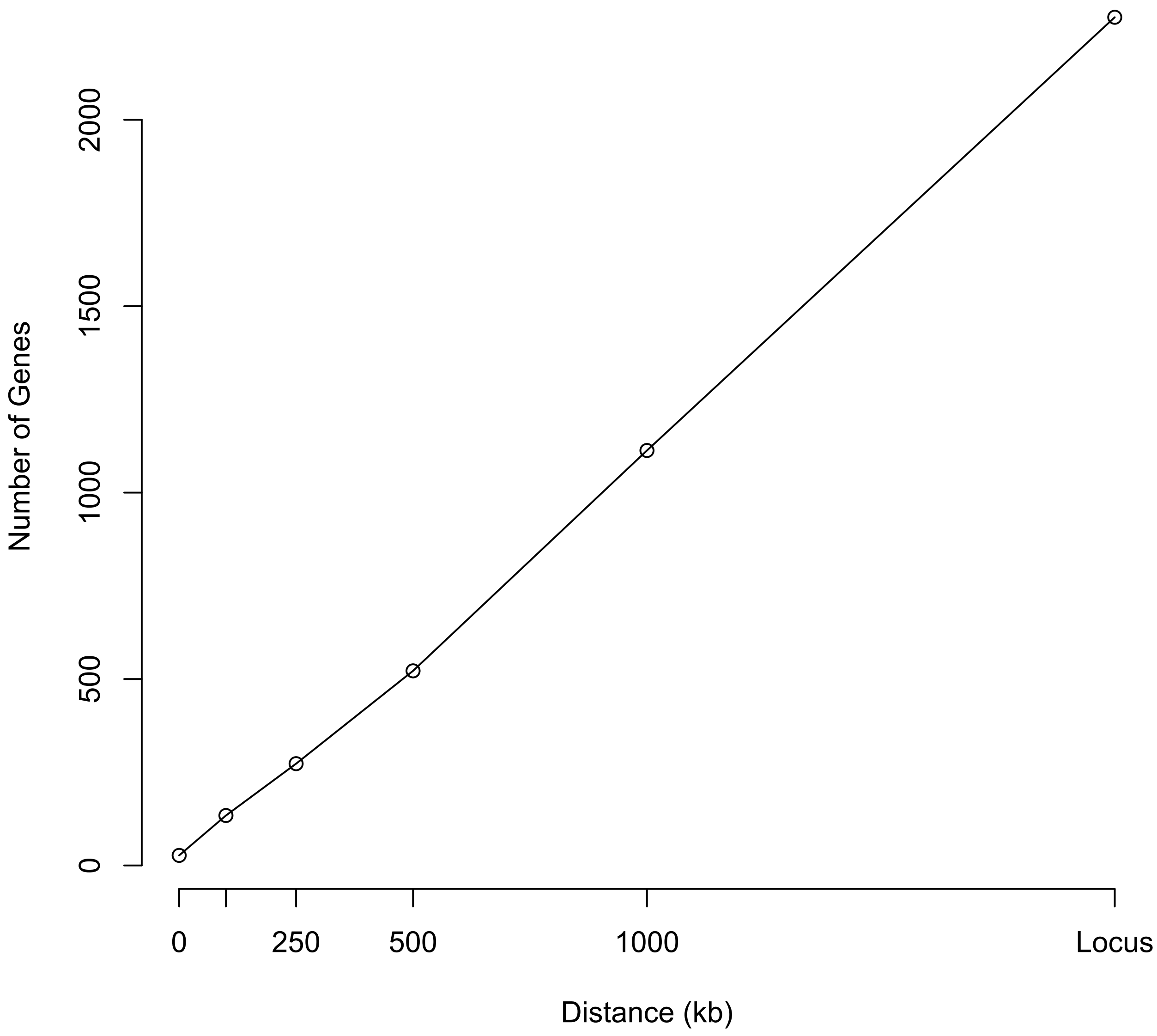

Number of Genes
0
500
1000
1500
2000
0
250
500
1000
Locus
Distance (kb)
